# Heparin and Direct Oral Anticoagulants have Different Effects on the Phases of Activation and Spatial Spread of Blood Coagulation

**DOI:** 10.1055/a-2516-7384

**Published:** 2025-02-17

**Authors:** Fazoil I. Ataullakhanov, Natalya M. Dashkevich, Ruzanna A. Ovsepyan, Tatiana A. Vuimo, Anna N. Balandina, Anna D. Kuprash, Dorzo-Cyren B. Ayusheev, Alexey I. Bernakevich, Elena I. Sinauridze

**Affiliations:** 1 Laboratory of Physiology and Biophysics of the Cell, Center for Theoretical Problems of Physicochemical Pharmacology, Russian Academy of Sciences, Moscow, Russia; 2 Physiology Department, Perelman School of Medicine, University of Pennsylvania, Philadelphia, Pennsylvania, United States; 3 Laboratory of Cell Hemostasis Mechanisms, Center for Theoretical Problems of Physicochemical Pharmacology, Russian Academy of Sciences, Moscow, Russia; 4 Scientific Department, Hematological Corporation HemaCore LLC, Moscow, Russia; 5 Laboratory of Blood Coagulation Regulation and Integral Phenotyping, Center for Theoretical Problems of Physicochemical Pharmacology, Russian Academy of Sciences, Moscow, Russia; 6 Laboratory of Cell Hemostasis and Thrombosis, Dmitry Rogachev National Medical Research Center of Pediatric Hematology, Oncology, and Immunology, Ministry of Healthcare, Moscow, Russia; 7 Department of Large Vessel Endoprosthetics, N.N. Priorov National Medical Research Center for Traumatology and Orthopedics, Ministry of Healthcare, Moscow, Russia; 8 Clinical and Diagnostic Laboratory, N.N. Priorov National Medical Research Center for Traumatology and Orthopedics, Ministry of Healthcare, Moscow, Russia; 9 Laboratory of Biophysics, Dmitry Rogachev National Medical Research Center of Pediatric Hematology, Oncology, and Immunology, Ministry of Healthcare, Moscow, Russia

**Keywords:** anticoagulants, blood coagulation, spatial clot growth rate, spatial thrombin distribution, Thrombodynamics-4D

## Abstract

**Background:**

Various reactions are involved in the phases of activation and further propagation of coagulation in space. The effects of different anticoagulants on these phases are unknown. Our aim was to study how different anticoagulants affect the activation and propagation phases of coagulation.

**Materials and Methods:**

Coagulation in the presence of low-molecular-weight heparin (nadroparin), and direct oral thrombin or factor Xa inhibitors (dabigatran and rivaroxaban, respectively) was studied in vitro and ex vivo via a global blood coagulation assay (Thrombodynamics-4D), which allows simultaneous analysis of thrombin activity in space and the clot growth rate. The ex vivo measurements were carried out in dynamics (8–9 days). The presence of asymptomatic thrombosis after 7 to 8 days of treatment was determined for each group of patients via ultrasound of the lower extremities.

**Results:**

All the tested anticoagulants inhibited thrombin generation but resulted in different patterns of thrombin spatial distribution and clot growth. The reversible inhibitors—dabigatran and rivaroxaban—inhibited the initiation phase of coagulation, while further clot growth was altered moderately. Irreversible nadroparin weakly affected the initiation phase of thrombin generation, but unlike dabigatran and rivaroxaban, it could completely suppress spatial thrombin propagation. Asymptomatic thrombosis was observed in 0%, 11%, and 29% of the patients in the nadroparin, dabigatran, and rivaroxaban groups, respectively.

**Conclusion:**

Antithrombin-dependent and independent inhibitors act differently on different phases of coagulation. High concentrations of dabigatran or rivaroxaban are insufficient to completely prevent fibrin clot growth, but even small amounts of heparin completely suppress this growth, due to factor IXa inhibition.

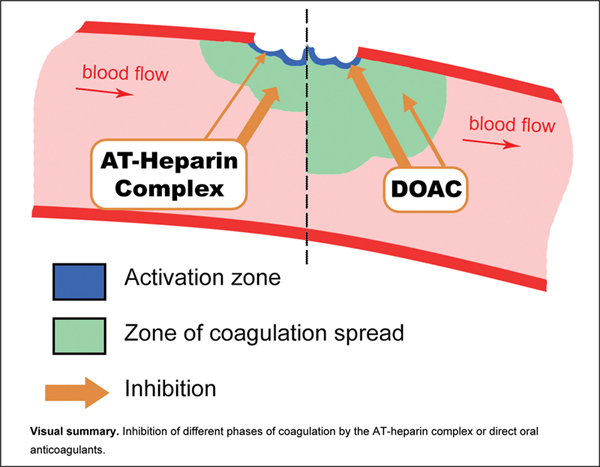

## Introduction

The formation of a hemostatic plug at the site of vessel injury is a result of complex interactions among the vessel wall, blood cells, and plasma coagulation proteins. 1 Typically, under physiological conditions, the initiation phase starts with the contact of blood with tissue factor (TF) on the cell surface at the site of endothelium damage, where thrombus growth begins. When a thrombus covers the site of endothelial damage (surface with TF), further work of the coagulation cascade continues on the surface of activated platelets and vesicles carrying phosphatidylserine. 2 3 4 5 6 At this stage, coagulation does not depend on the initial TF signal but depends mainly on the reaction of the intrinsic pathway (activation of factor XI by thrombin). 6 7 8 9 In some pathologies, the risk of thrombosis increases. For the treatment and prevention of thrombotic complications, coagulation inhibitors (anticoagulants) are used. The molecular mechanisms of anticoagulant action are well-characterized. The anticoagulants studied in this paper act either through an antithrombin (AT)-dependent mechanism (heparin), increasing to varying degrees the inhibition of several coagulation factors by AT or directly inhibiting one of the coagulation factors (thrombin or factor Xa [FXa]). It is assumed that the inhibition of individual procoagulant factors can have a more predictable effect than the inhibition of all serine proteases of the cascade by AT or antagonists of vitamin K. 10 11 Thrombin and FXa are necessary for clot formation at any of activation mechanisms (intrinsic or extrinsic). Thrombin is also important for the positive feedback loops of the cascade, which is responsible for FV, FVIII, and FXI activation, and further clot growth. 12 13 14 Thus, direct inhibition of these factors decreases their activity and can affect the activation and spread of coagulation.

Previously, to study coagulation, we developed the Thrombodynamics method, in which the coagulation activator is TF, localized on a solid surface and simulating a damaged vessel wall. This system is principally different from homogeneous systems with complete mixing of the activator and blood. 15 16 The idea of this experimental approach is the physical separation of the phases of coagulation initiation and clot growth, as occurs in living organisms. The addition of a thrombin-specific fluorogenic substrate to this system (Thrombodynamics-4D [TD-4D]) allowed us to monitor both fibrin clot formation and the generation and propagation of active thrombin in space simultaneously. The propagation of thrombin formation in this system is independent of TF and is determined by the positive feedback loop of the intrinsic pathway. 8

The thrombin generation test (TGT) is now widely used for the analysis of coagulation in various situations. 17 18 19 However, this test is performed in a homogeneous system with a fully mixed activator, which makes it less physiological. After the plasma is mixed with TF, the thrombin activity in the sample begins to increase rapidly. Then, a phase of inhibition follows. As a result, a peak appears on the calculated kinetic curve of active thrombin. In this system, fibrin formation occurs when only a small amount of thrombin is produced, 20 and most thrombin is generated after clot formation. However, within the framework of this approach, there is no answer to the question of why this excessive amount of thrombin is needed for blood clotting.

**Fig. 1:**
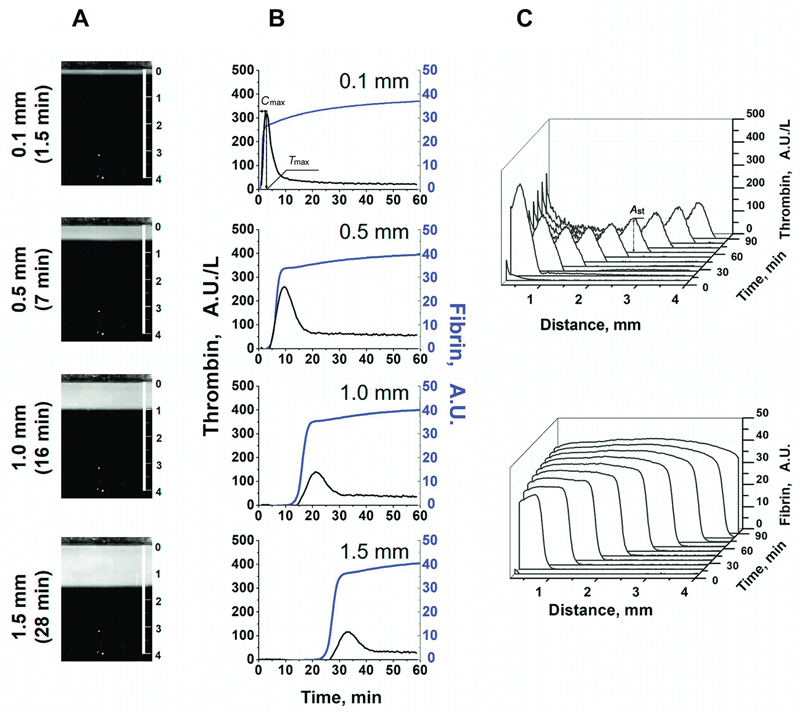
Propagation of thrombin and fibrin generation in space. ( **A** ) Images of growing fibrin clots at sequential time points (typical experiment). The clot size (in mm) is presented. The vertical scale bar on the right is 4 mm. ( **B** ) Peak thrombin generation (black line) and fibrin formation (blue line) at successive points reached by thrombin (only fibrin caused by thrombin formed at that particular time point is represented). *C*_max_ and *T*_max_ correspond to the maximum thrombin peak near the activator surface and the time to reach this concentration, respectively. ( **C** ) Two-dimensional representation of thrombin and fibrin propagation in space. *A*_st_ is the amplitude of the propagating thrombin peak 60 minutes after the activation of coagulation.

The spatial distribution of thrombin in normal plasma during clot growth was previously described. 8 Initially, thrombin is produced on the activating surface in direct contact with TF. Fibrin formation begins approximately 1 minute after activation ( Fig. 1A ). A fundamental feature of the blood coagulation system is that the further spread of thrombin occurs not through the diffusion of active thrombin from the site of activation but rather through the subsequent activation of the entire cascade at each point where a small amount of thrombin enters. This leads to self-supporting clot growth. 1 8 12 14

At each time point, a new thrombin peak is initiated. Thus, sequentially generated peaks of thrombin form a moving wave of thrombin, followed by fibrin formation ( Fig. 1B, C ). At each next time point, the thrombin activity curve has the same peak shape but with an increasing delay time. Fibrin formation at each point occurs at the very early stages of thrombin generation, which is in accordance with previously published data. The height of the thrombin peak decreases rapidly with increasing distance from an activator, as the influence of TF weakens. However, even if the TF is covered with 1 to 2 mm of fibrin, the clot continues growing. In this system, steady-state propagation of thrombin can be observed, 8 which means that thrombin supports its own propagation. 8 21 22 23 24 The goal of this work was to study the detailed effects of different types of anticoagulants on the spatial distribution of thrombin in the phases of coagulation activation and its further propagation, as well as the resulting effects of these anticoagulants on fibrin clot growth. We examined direct reversible inhibitors of FXa (rivaroxaban) and thrombin (dabigatran or dabigatran etexilate in ex vivo experiments), as well as low-molecular-weight heparin (LMWH; nadroparin) and unfractionated heparin (UFH; only in vitro), which irreversibly inhibit several coagulation factors, including FXa, FIXa, FXIa, and thrombin, in the presence of AT. Reversible coagulation inhibitors at therapeutic concentrations act on the activation phase of coagulation, increasing the time to thrombin appearance and the onset of fibrin clot formation, but cannot completely stop further clot growth. On the other hand, irreversible anticoagulants (nadroparin and UFH) affect the rates of clot growth and thrombin propagation in space but do not significantly affect the time of onset of thrombin and fibrin formation.

## Materials and Methods

### Donors and Patients

The effects of various anticoagulants on plasma coagulation were studied both in vitro and ex vivo. For in vitro experiments, blood from healthy donors ( *N* = 36) was used (16 males and 20 females aged 22–62 years). Donors did not receive any medication for 2 weeks prior to the study. Coagulation in vitro was studied in the presence of different concentrations of LMWH nadroparin, UFH, dabigatran, or rivaroxaban.

The ex vivo part of the study included 75 adult patients who underwent elective replacement of the hip or knee joints between February 2015 and June 2016 at the N.N. Priorov National Medical Research Center for Traumatology and Orthopedics, Moscow, Russia (25 males, 50 females, 24–80 years old, weighing 45–127 kg). After surgery, all patients were randomly divided into three groups and received various anticoagulant prophylactic therapies. Thirty-three patients received rivaroxaban, a direct inhibitor of FXa-10 mg once a day; 25 27 patients received dabigatran etexilate, which turns into a direct thrombin inhibitor (DTI) dabigatran—220 mg once a day; 26 and 15 patients received LMWH nadroparin twice a day at a dosage of 2,850 or 5,700 anti-Xa IU in the morning and 2,850 anti-Xa IU in the evening (depending on the patient's weight), which corresponds to a high prophylactic dose of nadroparin. 27 The male-female ratio, mean age, and mean body weight did not significantly differ among the three groups (one-way two-tailed analysis of variance [ANOVA], *p* < 0.05; Supplementary Table S2 [available in the online version only]).

Maximum plasma concentrations of all anticoagulants studied (Amax) after administration of the used prophylactic or maximum therapeutic doses of these drugs were not measured but were calculated based on literature data 28 29 30 31 32 33 (see Supplementary Material, sections 1.2 and 1.3 [available in the online version only]). In particular, the concentration of nadroparin was calculated based on data from a study, 28 which showed that when it was transfused at a dose of 43.1 anti-Xa IU/kg, the Amax was 0.252 IU/kg. In the case of our patients, Amax was 95 anti-Xa IU/kg, because they obtained the nadroparin dose 5,700 anti-Xa IU at the weight of 60 kg but less than 90 kg, or 8,550 anti-Xa IU at ≥90 kg. Given that for LMWH, the dose of the drug is directly proportional to its maximum plasma concentration, we deduced that a dose of 95 IU/kg should result in Amax = 0.58 IU/mL (∼0.6 IU/mL).

### Thrombodynamics-4D Test

According to reference, 22 simultaneous measurements of light scattering (at λ = 625 nm) and fluorescence of 7-amine-4-methylcoumarin (AMC), which is a product of thrombin-specific fluorogenic substrate hydrolysis by thrombin (at λ _excitation_ = 365 nm; λ _emission_ = 440 nm) were performed using a Thrombodynamics® Analyzer T2T for 90 minutes at 37 °C. Coagulation activators (plates coated at one end with immobilized TF) were prepared via a previously described method. 34 Measurements were performed in platelet-free plasma ( Supplementary Material , section 2.2 [available in the online version only]). All rules for the preanalytical preparation of plasma samples were standard, as for all coagulation assays. 35 The results were recorded via a charge-coupled device (camera) every 6 seconds and processed using an automated calculation algorithm via software specially developed by the manufacturers. Images from red and UV light were processed similarly. To obtain light scattering or AMC fluorescence intensity profiles, the light intensity along the line perpendicular to the activator surface was measured for each frame. The principle of calculating active thrombin concentrations was similar to that used in the TGT but took into account the diffusion of AMC in space. 8 22 36

Briefly, activation of coagulation results in the formation of thrombin, which hydrolyzes the thrombin-specific fluorogenic substrate present in plasma. The setup records the emerging fluorescence of the product of this hydrolysis (AMC) over the entire area of the sample every 6 seconds. The result is a set of AMC profiles, each of which characterizes the distribution of AMC in space at the corresponding moment in time. The thrombin activity at each moment in time at each point in space is proportional to the rate of AMC accumulation. This rate can be obtained by differentiating the recorded AMC profiles. It should also be taken into account that the concentration of AMC at any given moment in time consists not only of AMC formed as a result of the substrate hydrolysis reaction by thrombin but also of AMC that has ended up at this point due to its diffusion. Thus, to calculate the activity of thrombin, it is necessary to use the reaction–diffusion equation (1) :



where *D*_AMC_ is the diffusion coefficient of AMC, [S] and [IIa] are the concentrations of the substrate and thrombin, respectively, and *k*_cat_ and *K*_M_ are the catalytic constant and Michaelis constant for the reaction of the substrate hydrolysis by thrombin, which obeys the Michaelis–Menten equation (these constants are known). From this equation, by solving the inverse problem, one can calculate the concentration of active thrombin at each moment in time at each point in space using equation (2) :



It should be noted that the final calculation also takes into account the fact that 67% of the resulting AMC binds to plasma albumin, resulting in a decrease in its diffusion rate and that the presence of fibrin also affects the fluorescence of AMC. All this was shown in separate experiments. 8 We do not provide, here, a detailed description of the calculation of the thrombin concentration from the measured AMС fluorescence (with all necessary corrections) due to space limitations, and because this was described in great detail in the supplementary material of the work by Dashkevich et al., 8 as well as in the works. 19 22 36

The following TD-4D parameters were measured.

*T*_lag_ is the time between coagulation activation upon contact with TF and the beginning of fibrin formation on the activating surface;

*V*_i_ and *V*_st_ are the initial and steady-state clot growth rates measured at intervals of 2 to 6 minutes and 15 to 25 minutes after *T*_lag_ , respectively;

*C*_max_ and *T*_max_ are the height of the thrombin peak near the activator and the time to reach this peak, respectively;

*A*_st_ is the height of the thrombin peak running in space 60 minutes after activation;

*V*_t_ and *V*_f_ are the rates of thrombin or fibrin propagation, respectively, in the interval of 45 to 55 minutes, which were shown to be equal ( Supplementary Fig. S1 [available in the online version only]);

*T*_sp_ is the time at which spontaneous clots appear in the sample. The presence of such clots indicates plasma hypercoagulation. 5

For a detailed description of the reagents, sample preparation, method of TD-4D and its parameters, as well as normal values, reproducibility, and interindividual variability of these parameters, see Supplementary Material, section 2 and Supplementary Table S1 (available in the online version only).

### Ex vivo Study Design

The following exclusion criteria were used for the patient selection: (1) age less than 18 years; (2) presence of hematological diseases; (3) taking anticoagulants within 2 weeks before hospitalization; (4) pregnancy; and (5) refusion to participate in the study. No selected patients were withdrawn from the study during treatment.

The TD-4D test was performed at the following checkpoints within 8 to 9 days after surgery.

For dabigatran and rivarixaban

Point 1: one hour before the operation (Day 1).
Points 2 and 3: the morning following the day after surgery, before and 2 to 3 hours after the first dose of anticoagulant, respectively (Day 2).
Points 4 and 5: in the morning before and 2 to 3 hours after the second dose of anticoagulant, respectively (Day 3).
Point 6: Day 7 (or 8), 2 to 3 hours after the scheduled anticoagulant intake.
Point 7: Day 8 (or 9), in the morning before the regular dose of anticoagulant.

For LMWH, which was administered two times a day subcutaneously, all samples were taken on analogous days in the morning (before or 2–3 hours after administration of the appropriate dose of anticoagulant).

A diagram of the anticoagulant administration times and the measurement points corresponding to its maximum and minimum concentrations is shown in Supplementary Fig. S3 (available in the online version only).

On days 7 to 8 of hospitalization, the patients underwent an ultrasound of the lower extremity veins to detect thrombotic complications in the postoperative period. The data are presented in Supplementary Table S2 (available in the online version only).

### Data Analysis

The normality of the distributions of all the parameters was examined via the D'Agostino-Pearson test (program MedCalc, version 14.12; MedCalc Statistical Software bvba, Ostend, Belgium).

The in vitro inhibitory effect of different concentrations of each anticoagulant on TD-4D parameters was calculated as a percentage relative to the values of these parameters in the system without inhibitor and averaged overall relevant samples.

The TD-4D test parameters were also compared in several pairs of adjacent points with the minimum and maximum effect of the drug (before and 3 hours after taking the anticoagulant). For this, the paired Wilcoxon's *t* -test (Wilcoxon signed-rank test) was used together with the Holm correction for multiple comparisons, which was performed using the “stats” package in R (v 4.4.0). Differences were considered significant at *p* -values <0.05.

## Results

### Effect of Anticoagulants on Clot Growth and Thrombin Distribution In Vitro

In vitro experiments avoid changes in the concentrations of added anticoagulants in the sample, which is possible in ex vivo experiments because of the distribution of the drug in the body and the different rates of its elimination in different patients.

**Fig. 2:**
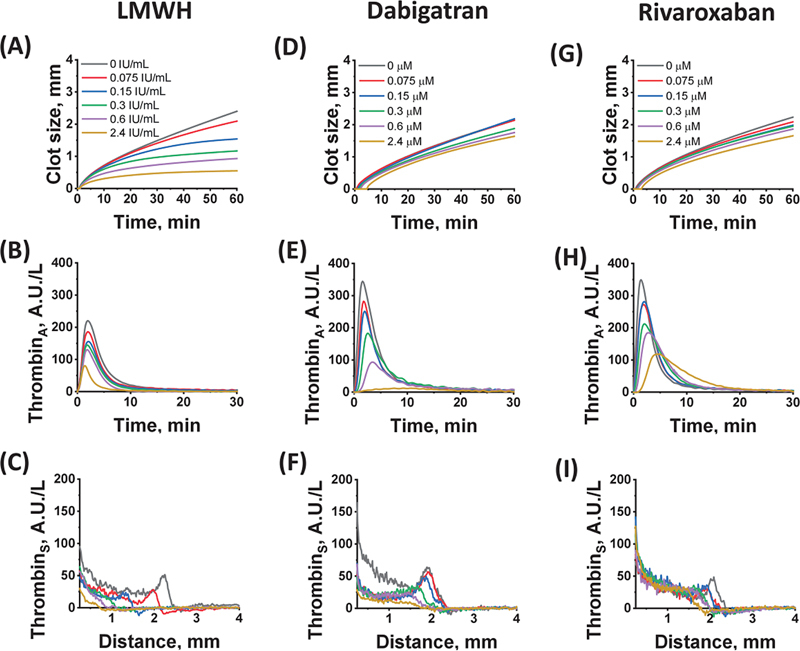
Typical examples of the effects of various anticoagulants in vitro on the size of the fibrin clot, the thrombin distribution near the activator (Thrombin _A_ ), and its spatial distribution at 60 minutes after activation (Thrombin _S_ ) in the plasma of individual donors. **A-C:** LMWH nadroparin, **F-D:** dabigatran, and **G-I:** rivaroxaban. Curves corresponding to different concentrations of anticoagulants are presented (see panels **A** , **D,** and **G** ). LMWH, low-molecular-weight heparin.

To study the effects of LMWH, UFH, dabigatran, and rivaroxaban on different phases of coagulation, increasing concentrations of these anticoagulants were added to the plasma of different donors. The effects of these concentrations on all the TD-4D parameters were compared. Figure 2 shows typical curves for some TD-4D parameters obtained for individual donors in the presence of various concentrations of nadroparin, dabigatran, and rivaroxaban.

**Fig. 3:**
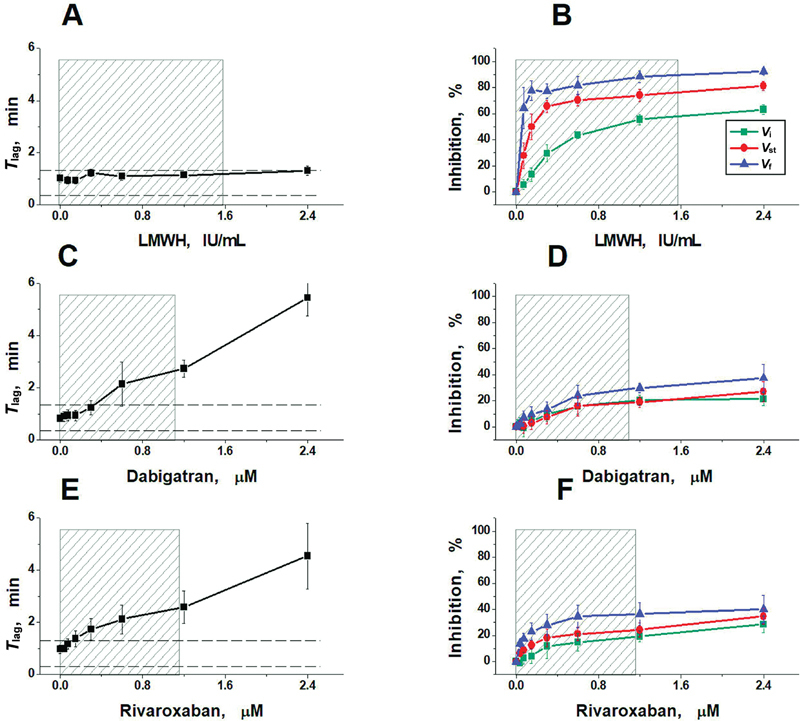
Effects of LMWH nadroparin ( **A, B** ), dabigatran ( **C, D** ), and rivaroxaban ( **E, F** ) on fibrin formation parameters *T*_lag_ , *V*_i_ , *V*_st_ , and *V*_f_ . The mean values ± standard deviations are presented. *N* = 8 for each group. The shaded areas represent the plasma concentrations that can be obtained for each anticoagulant during therapeutic or prophylactic treatment (see Supplementary Material, section 1.3 , available in the online version only). The horizontal dashed lines represent the normal range for the *T*_lag_ value. LMWH, low-molecular-weight heparin.

**Fig. 4:**
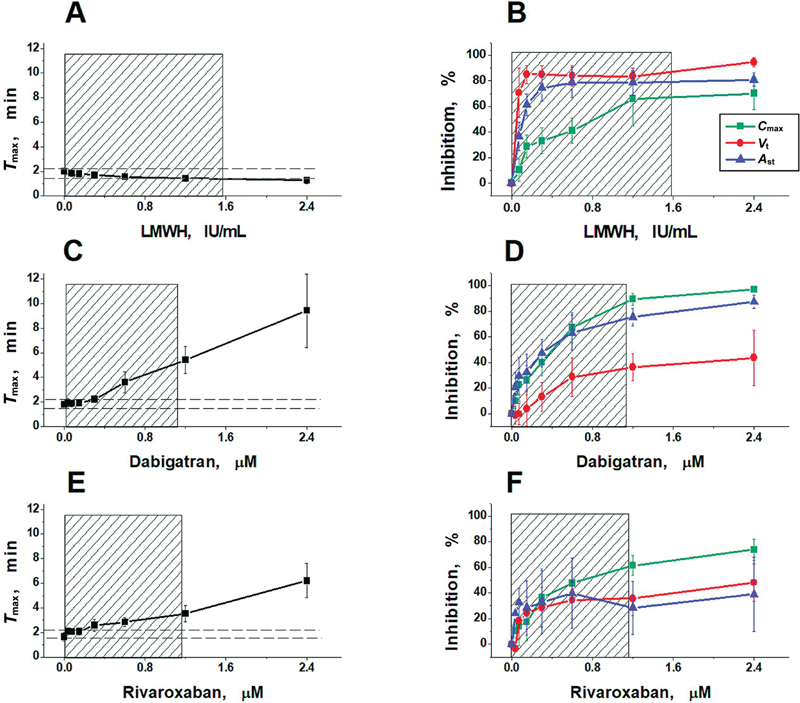
Effects of LMWH nadroparin ( **A, B** ), dabigatran ( **C, D** ), and rivaroxaban ( **E, F** ) on thrombin generation parameters *T*_max_ , *C*_max_ , *V*_t_ , and *A*_st_ . The mean values ± standard deviations are presented. *N* = 8 for each group. The shaded areas represent the plasma concentrations that can be obtained for each anticoagulant during therapeutic or prophylactic treatment (see Supplementary Material, section 1.3 , available in the online version only). The horizontal dashed lines represent the normal range for the *T*_max_ value. LMWH, low-molecular-weight heparin.

The averaged results of all experiments in vitro, obtained for the main parameters of the clot growth and spatial thrombin generation, are presented in Figs. 3 and 4 , and Supplementary Fig. S2 (available in the online version only).

### Nadroparin

The TD-4D parameters in the presence of different concentrations of nadroparin are presented in Figs. 3A, B and 4A, B . Increasing the LMWH concentration to 2.4 anti-Xa IU/mL did not increase the delay in the onset of clot formation ( *T*_lag_ ; Fig. 3A ). In contrast, spatial clot growth slowed greatly. In the range of nadroparin concentrations of 0.3 to 2.4 anti-Xa IU/mL, an almost complete cessation of clot growth was observed for *V*_f_ and *V*_st_ ( Fig. 3B ). The sensitivity of various parameters describing the spatial rate of clot growth to nadroparin increased with increasing distance from the coagulation-activating surface ( *V*_i_ < *V*_st_ < *V*_f_ ). The clot growth rate 45 to 55 minutes after activation was most sensitive to LMWH. It decreased by about 80% at the LMWH concentration of 0.15 anti-Xa IU/mL and then remained almost unchanged. In contrast, the decrease in the initial clot growth rate ( *V*_i_ ) with increasing LMWH concentration was the slowest, but this rate continued to gradually decrease to a heparin concentration of 2.4 anti-Xa IU/mL ( Fig. 3B ). Fibrin clot formation was still detectable at the nadroparin concentration of 4.8 anti-Xa IU/mL as a thin layer on the activator colocalized with residual thrombin activity ( Supplementary Fig. S4A, B [available in the online version only]), but a further increase in the heparin concentration led to complete inhibition of clot formation.

Nadroparin moderately affected the maximum value of the thrombin peak near the activator surface ( *C*_max_ ) but did not affect the time to reach this maximum peak ( *T*_max_ ; Fig. 4A, B ). It also significantly inhibited other parameters of the thrombin propagation phase ( *V*_t_ and *A*_st_ ; Fig. 4B ).

The most sensitive parameter to LMWH was *V*_t_ . Its value decreased by approximately 85% at a nadroparin concentration of 0.15 anti-Xa IU/mL, whereas *A*_st_ and *C*_max_ at the same LMWH concentration were reduced by approximately 61.6% and 28.8%, respectively. Thus, the action of LMWH rapidly decreased the moving thrombin peak, simultaneously with the inhibition of clot growth. At nadroparin concentrations above 0.15 anti-Xa IU/mL, thrombin generation was still observed, but its propagation rate was significantly reduced. The thrombin wave disappeared, and the thrombin distribution profile changed to diffusional ( Fig. 2C ).

Quite similar results were obtained in in vitro experiments with UFH ( Supplementary Fig. S2 [available in the online version only]).

#### Dabigatran and Rivaroxaban

Compared with nadroparin, direct oral anticoagulants (DOACs; direct thrombin or FXa inhibitors) had a significantly weaker effect on fibrin clot propagation ( Fig. 3D, F ), although the onset of fibrin formation ( *T*_lag_ ) slowed with increasing in their concentrations ( Fig. 3C, E ). As in the case of LMWH, the rate *V*_f_ was the most sensitive to dabigatran or rivaroxaban concentration, but it decreased only 20 to 30% from the initial value within the therapeutic range of their concentrations. Increasing the concentration of both DOACs to 2.4 μM resulted in an additional reduction of only 5 to 10%.

DOACs essentially affected the thrombin peak *C*_max_ at the activating surface ( Fig. 4D, F ). The peak height decreased, and the time to peak ( *T*_max_ ) was prolonged ( Fig. 4C, E ), resulting in a delay in fibrin formation. The height of the moving thrombin peak *A*_st_ decreased synchronously with *C*_max_ near the activator. An increase in the concentration of dabigatran above 2 μM led to the transformation of the mobile thrombin peak into a plateau with a sharp decrease in the thrombin concentration at its border ( Fig. 2F ). Rivaroxaban also changed the shape of the thrombin peak, lengthening the thrombin inhibition process, which led to a widening of the peak ( Fig. 2H, I ). Unlike LMWH, with increasing rivaroxaban concentration, the thrombin peak transformed into a plateau rather than into a diffusional distribution ( Fig. 2I ). As with dabigatran, this plateau could still spread in space and control the formation of a fibrin clot.

The differences between LMWH and the DOACs were also confirmed by in vitro experiments where the anticoagulant concentrations were much higher than therapeutic ones. AT-dependent nadroparin at a concentration of 4.8 anti-Xa IU/mL reduced thrombin formation and spread ( Supplementary Fig. S4B [available in the online version only]), as well as the spatial clot growth rate ( Supplementary Fig. S4A [available in the online version only]) to almost zero. On the other hand, reversible AT-independent anticoagulants dabigatran and rivaroxaban also reduced the spatial clot growth but were significantly weaker than heparin. They suppressed thrombin production more than clot growth ( Supplementary Fig. S4D, F [available in the online version only]), especially in the case of dabigatran ( Supplementary Fig. S4C, D [available in the online version only]). The clot continued to grow even at dabigatran concentrations of 5, 9.6, and 20 μМ ( Supplementary Figs. S4C and S5E, F [available in the online version only]). A similar pattern was also observed in the presence of a high (9.6 μM) concentration of rivaroxaban ( Supplementary Fig. S4E [available in the online version only]).

These data indicate that strong specific inhibition of thrombin does not prevent the formation and spread of fibrin in space completely even at very high DOAC concentrations.

### Effects of Various Anticoagulants on Ex Vivo Coagulation

To confirm the in vitro data on the effect of various anticoagulants on the activation and propagation phases of coagulation, plasma samples from patients after elective knee or hip replacement who received anticoagulant prophylaxis in the postoperative period were studied. The study lasted 8 to 9 days while the patients were in the clinic and included 75 patients who were divided into three groups treated with LMWH nadroparin ( *N* = 15), dabigatran etexilate, dabigatran prodrug ( *N* = 27), and rivaroxaban ( *N* = 33). Prophylactic doses of each drug were used, which were approximately equal in efficacy. 25 26 27 The study design, patients, and doses are described in the “Materials and methods” section and Supplementary Material, sections 1, 3, 4 (available in the online version only).

The results obtained ex vivo for various anticoagulants confirmed the findings of the in vitro experiments.

#### Nadroparin Prophylactic Therapy

**Fig. 5:**
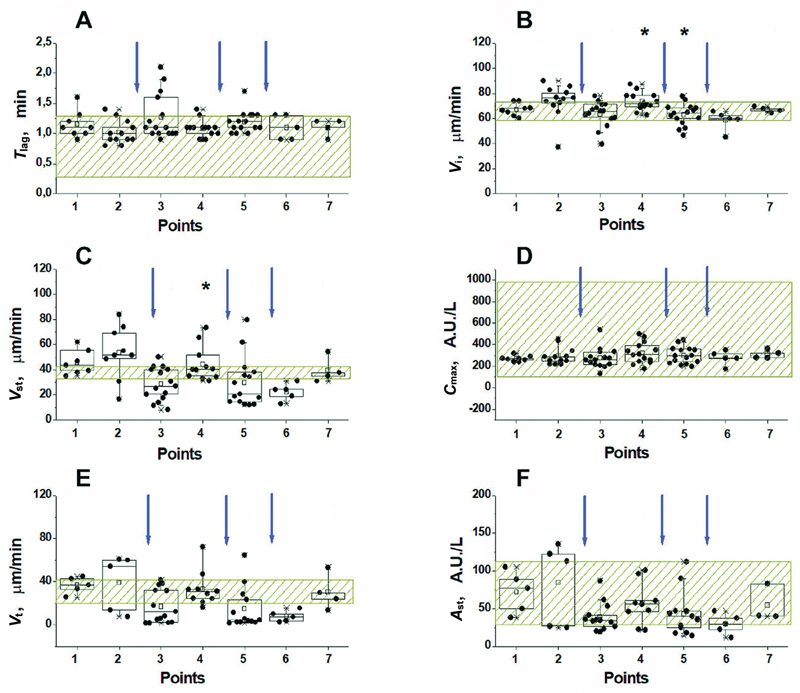
Effect of nadroparin on TD-4D parameters at different time points before and after surgery (see “Materials and methods” section, and Supplementary Fig. S3 [available in the online version only]). The values of the lag period before the fibrin appearance ( **A** ), the initial ( **B** ) and stationary ( **C** ) clot growth rates, the maximum thrombin concentration near the activator ( **D** ), the rate of thrombin propagation in the time interval 45 to 55 minutes after activation ( **E** ), and the height of the thrombin peak 60 minutes after activation ( **F** ) are presented. The shaded zone corresponds to the range of normal values for each parameter. The arrows indicate the time of nadroparin administration. Box sizes correspond to the range from 25th to 75th percentiles of all the measured values. The medians are represented by horizontal lines, the length of the whiskers corresponds to the range from the 5th to the 95th, and the “ × ” signs from 1st to 99th percentiles of all the measured values. *The value is significantly different from this value at the previous point (Wilcoxon signed-rank test with the Holm correction for multiple comparisons, *p* < 0.05). TD-4D, Thrombodynamics-4D.

All the results obtained for patients treated with nadroparin are presented in Fig. 5 . To avoid overloading the figure, only the significance of differences between adjacent points with maximum and minimum drug effects is presented. These are pairs of points: 3/2, 3/4, 5/4, and 6/7. The *p* -values for the significance of the differences between all studied pairs of points for all anticoagulants are presented in Supplementary Table S3 (available in the online version only).

Nadroparin did not result in a significant change in *T*_lag_ ( Fig. 5A ) but did result in a significant decrease in initial and especially stationary rates of thrombus growth below normal in most patients at the peak of LMWH action (points 3, 5, and 6). At the end of the nadroparin dose action (12 hours after administration), the values of these parameters returned to the hypercoagulation (point 4) and normal coagulation (point 7) regions. Trends in the parameters characterizing thrombin generation at different points of the study are clearly visible in Fig. 5D–F . It turned out that the maximum concentration of thrombin near the activator ( *C*_max_ ) did not differ significantly at different points of the study ( Fig. 5D ). This also applies to *A*_st_ values, for which there was a decreasing trend at the maximum nadroparin action (points 3, 5 and 6). However, because of large variations, these changes were not reliable, and the value of the parameter *A*_st_ for all points remained within the normal range ( Fig. 5F ). *V*_t_ and *A*_st_ values, always decreased at the points of maximum drug action (points 3, 5, and 6), but increased again by the end of the drug dose action (points 4 and 7). However, the differences between these values were significant only after the second and subsequent administrations, although the trend *V*_t_ after the first administration was the same ( Fig. 5E ). Thus, the significance of the differences between the maximum and minimum points of nadroparin dose action was more pronounced for the *V*_t_ parameter than for *A*_st_ .

The reduction in hypercoagulability after LMWH administration was also confirmed by a decrease in the percentage of samples with spontaneous clots observed ( Supplementary Figs. S6 and S7 [available in the online version only]). The number of spontaneous clots in the samples after nadroparin administration was reduced at the peak of drug action (points 3, and 5), but increased again at the end of its action (point 4)

#### Thromboprophylaxis with Direct Oral Anticoagulants

**Fig. 6:**
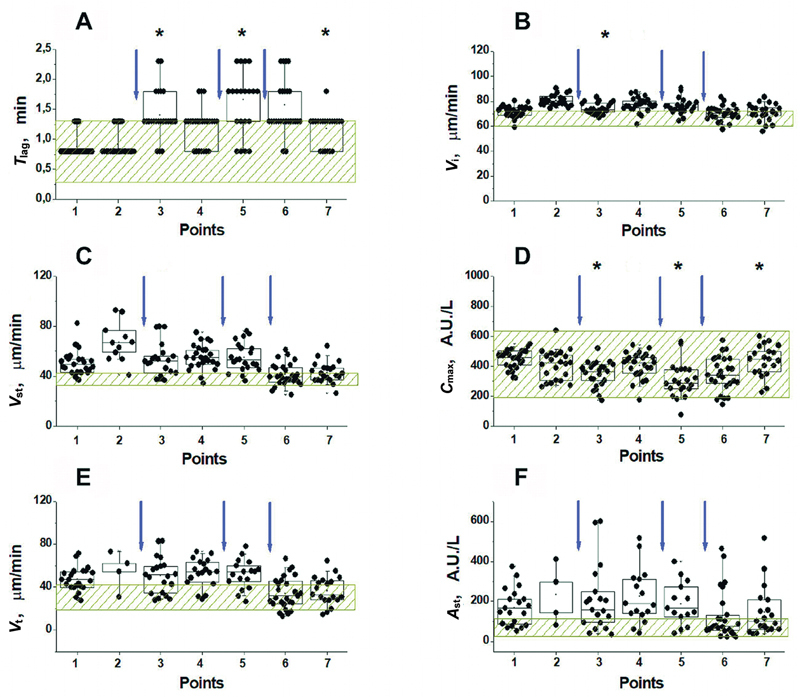
Effect of dabigatran etexilate on TD-4D parameters at different time points before and after surgery (see “Materials and methods” section, and Supplementary Fig. S3 [available in the online version only]). The values of the lag period before the fibrin appearance ( **A** ), the initial ( **B** ) and stationary ( **C** ) clot growth rates, the maximum thrombin concentration near the activator ( **D** ), the rate of thrombin propagation in the time interval 45 to 55 minutes after activation ( **E** ), and the height of the thrombin peak 60 minutes after activation ( **F** ) are presented. The shaded zone corresponds to the range of normal values for each parameter. The arrows indicate the time of drug administration. Box sizes correspond to the range from 25th to 75th percentiles of all the measured values. The medians are represented by horizontal lines, the length of the whiskers corresponds to the range from the 5th to 95th, and the “ × ” signs from 1st to 99th percentiles of all the measured values. * The value is significantly different from this value at the previous point (Wilcoxon signed-rank test with the Holm correction for multiple comparisons, *p* < 0.05). TD-4D, Thrombodynamics-4D.

**Fig. 7:**
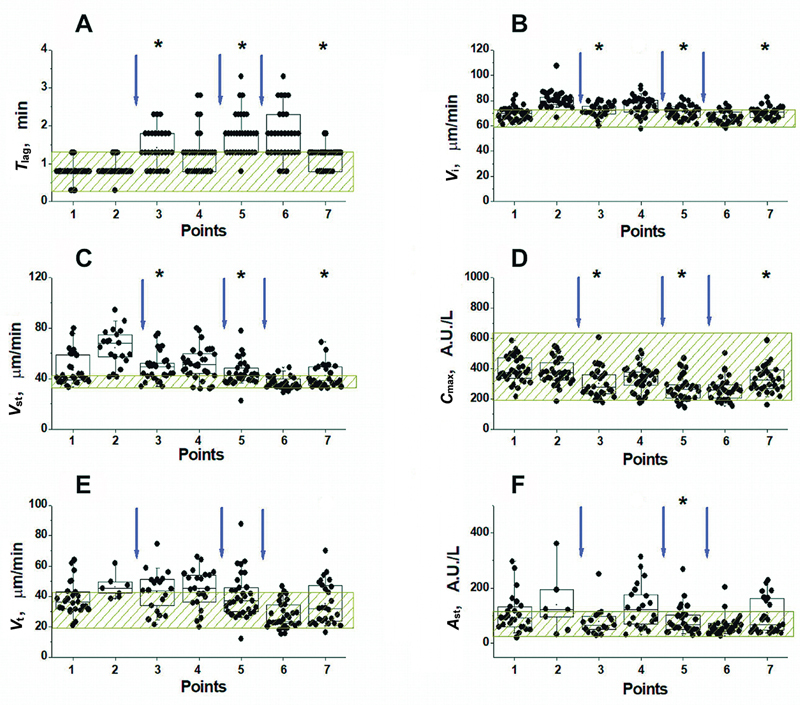
Effect of rivaroxaban on TD-4D parameters at different time points before and after surgery (see “Materials and methods” section and Supplementary Fig. S3 [available in the online version only]). The values of the lag period before the fibrin appearance ( **A** ), the initial ( **B** ) and stationary ( **C** ) clot growth rates, the maximum thrombin concentration near the activator ( **D** ), the rate of thrombin propagation in the time interval 45 to 55 minutes after activation ( **E** ), and the height of the thrombin peak 60 minutes after activation ( **F** ) are presented. The shaded zone corresponds to the range of normal values for each parameter. The arrows indicate the time of drug administration. Box sizes correspond to the range from 25th to 75th percentiles of all the measured values. The medians are represented by horizontal lines, the length of the whiskers corresponds to the range from the 5th to 95th, and the “ × ” signs from 1st to 99th percentiles of all the measured values. *The value is significantly different from this value at the previous point (Wilcoxon signed-rank test with the Holm correction for multiple comparisons, *p* < 0.05). TD-4D, Thrombodynamics-4D.

The second and third groups of patients received DOACs. These were dabigatran etexilate, a prodrug that quickly turns in the body into a direct reversible thrombin inhibitor dabigatran ( *N* = 27), and rivaroxaban, a reversible inhibitor of FXa ( *N* = 33). Changes in TD-4D parameters at different time points in all patients receiving these anticoagulants are shown in Figs. 6 and 7 , respectively. Unlike LMWH, these DOACs cause a significant increase in *T*_lag_ ( Figs. 6A and 7A ) and a decrease in *C*_max_ ( Figs. 6D and 7D ) after each dose of the drug (points 3 and 5). Although the amplitude of thrombin near the activator changed significantly after the administration of dabigatran etexilate or rivaroxaban, almost all *C*_max_ values remained within the normal range ( Figs. 6D and 7D ).

The clot growth rates ( *V*_i_ and *V*_st_ ) also decreased after taking the drugs, but unlike rivaroxaban, where the decrease was significant after each dose of the drug, dabigatran etexilate had the same general trend, but significantly reduced *V*_i_ only after the first and second doses, and *V*_st_ only after the first dose of the drug.

Measures characterizing the distribution of thrombin ( *V*_t_ and *A*_st_ ) showed a weak downward trend at the points of maximum effect of both drugs. However, for dabigatran etexilate, this effect was unreliable, and in the case of rivaroxaban, it was reliable only after the first dose of the drug for *V*_t_ , and after the first and second doses of the drug for *A*_st_ . Importantly, after a week of taking either of these two drugs, the difference between the parameters at the peak and the end of the drug dose action was erased, that is, almost all the parameters (except *T*_lag_ ) were within the normal range. Thus, after approximately 1 week, hemostasis from postoperative hypercoagulation returned to normocoagulation in most patients. We call hypercoagulation a condition in which any of the indicators characterizing the intensity of blood clotting shows an increase in this intensity that goes beyond the normal values.

### Comparison of the Effects of Different Anticoagulants on Thrombodynamics-4D Parameters In Vitro and Ex Vivo

**Fig. 8:**
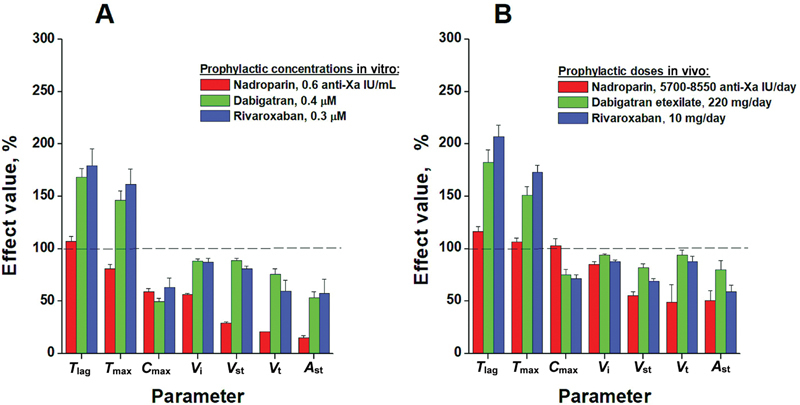
Comparison of the effect of various anticoagulants on TD-4D parameters characterizing fibrin distribution ( *T*_lag_ , *V*_i_ , *V*_st_ ) and thrombin formation ( *T*_max_ , *C*_max_ , *V*_t_ , and *A*_st_ ) in in vitro and ex vivo experiments. Effect values are presented as a percentage of the corresponding values in the absence of anticoagulants. The mean values and standard errors of the means are presented. ( **A** ) Changes in TD-4D parameters in in vitro experiments. To compare in vitro and ex vivo experiments, in vitro anticoagulant concentrations similar to those obtained ex vivo for the studied prophylactic doses of anticoagulants were used (see calculation in Supplementary Material, section 1.2 [available in the online version only]). They were as follows: LMWH = 0.6 anti-Xa IU/mL, dabigatran = 0.4 μM, and rivaroxaban = 0.3 μM ( *N* = 8 for each group). ( **B** ) TD-4D parameters in ex vivo experiments at point 5 (on the third day of the experiment, 3 hours after the administration of the corresponding anticoagulant) in relation to point 2 (after surgery but before the anticoagulant administration) in patients treated with LMWH nadroparin (5,700–8,550 anti-Xa IU/day, *N* = 15), dabigatran etexilate (220 mg/day, *N* = 27), or rivaroxaban (10 mg/day, *N* = 33). LMWH, low-molecular-weight heparin; TD-4D, Thrombodynamics-4D.

The mean changes in various TD-4D parameters (in percentages) relative to conditions without anticoagulants were calculated for in vitro and ex vivo experiments at fixed anticoagulant concentrations ( Fig. 8 ). To calculate parameters in in vitro experiments, concentrations close to those obtained in ex vivo experiments were used (0.6 anti-Xa IU/ml, 0.4 μM and 0.3 μM for LMWH, dabigatran, and rivaroxaban, respectively; Fig. 8A ). Calculations for ex vivo experiments were performed using the anticoagulant concentrations obtained in this study during patient prophylaxis. For this, the values at point 5 (after the second or third dose of anticoagulant for DOACs or LMWH, respectively) were calculated as a percentage of the values at the corresponding point 2 (after operation, but before anticoagulant administration; Fig. 8B ). The *V*_f_ values are not shown in Fig. 8 , because, according to Supplementary Fig. S1 (available in the online version only), they are equal to the *V*_t_ values.

For all anticoagulants in vitro, the degree of inhibition of clot growth in space increased with increasing distance from the activating surface ( *V*_i_ < *V*_st_ < *V*_f_ ). The levels of inhibition of all these rates were almost the same for both DOACs, but higher for LMWH ( Fig. 3B, D, F ). This was also true for similar parameters in ex vivo experiments (except for *V*_i_ , where inhibition was similar for all anticoagulants; Fig. 8B ). The parameters characterizing thrombin generation were fundamentally different for LMWH and DOACs (AT-dependent and independent inhibitors of thrombin and FXa, respectively). In the case of LMWH, inhibition was reduced in the following order: *V*_t_ > *A*_st_ > *C*_max_ ( Fig. 4B ), whereas for dabigatran and rivaroxaban, the decreasing order was reversed: *C*_max_ > *A*_st_ > *V*_t_ ( Fig. 4D, F ). Thus, the most and least sensitive parameters to DOAC were the concentration of thrombin near the activator ( *C*_max_ ) and the rate of thrombin propagation away from the activator ( *V*_t_ ), respectively. Moreover, despite a comparable decrease in *C*_max_ for in vitro experiments, the effects of the studied anticoagulants on *V*_t_ were qualitatively different, which indicates that thrombin activity does not fully determine this difference and, accordingly, the clot growth rate ( Fig. 8A ). A qualitatively similar pattern was also observed in ex vivo experiments.

Thus, the results obtained ex vivo were fully qualitatively consistent with the in vitro experimental data ( Fig. 8 ).

## Discussion

Currently, DOACs are considered good replacements for heparins, for the treatment and prevention of thrombotic complications. However, to date, facts are gradually accumulating, indicating that not only bleeding but also hypercoagulation disorders are possible with the use of DOACs. It has been shown 37 38 39 40 41 42 that when UFH or DTIs, dabigatran, ximelagatran, lepirudin, etc., are discontinued, a relapse of hypercoagulation may occur. The authors explain this by the fact that thrombin can both activate coagulation and inhibit it by activating the protein C system and the formation of prostacyclin. 39 40 41 42 In addition, in a rat model of disseminated intravascular coagulation, low doses of melagatran have been shown to increase intravascular coagulation by increasing thrombin formation. 38 FXa inhibitors are not thought to have a similar effect because they do not affect the protein C system. However, in this study, we showed that after 1 week of treatment with the studied anticoagulants, asymptomatic deep vein thrombosis was more common in patients receiving the FXa inhibitor rivaroxaban (29%), less common in patients receiving dabigatran etexilate (11%), and was absent from the nadroparin group ( Supplementary Table S2 [available in the online version only]).

No symptomatic thrombotic events were observed in the patients included in the study. In all cases of asymptomatic thrombosis, ultrasound signs of occlusive distal thrombosis of the vein soleus were observed, predominantly at the level of the upper and middle third ( Supplementary Table S2 [available in the online version only]). Based on the available data, we cannot reliably state how the number of asymptomatic thromboses in these groups quantitatively correlates. This is due to the small number of patients in each group, especially those receiving nadroparin. The only thing we are ready to state is that thromboses occurred in both groups of patients receiving DOACs. This result differs from data previously described in the literature and deserves special discussion.

Heparins irreversibly inhibit thrombin, FXa, FIXa, FXIa, and XIIa, enhancing AT activity, especially in the case of FIXa (about 10,000 or 100,000 times for nadroparin and UFH, respectively). 43 DOACs specifically inhibit only thrombin or FXa.

For the clot to begin to grow, the first small concentrations of thrombin must appear, which activate factors V and VIII, after which clotting accelerates explosively. At that, the concentration of the initially formed thrombin must exceed a certain threshold, since otherwise it will be completely inhibited by natural anticoagulants (AT, α _2_ -macroglobulin, etc.). 44 The time required for the formation of the thrombin concentration required to start accelerated coagulation determines the value of the clotting delay period ( *T*_lag_ ). In our study, *T*_lag_ values changed very slightly in the presence of heparins (at physiologically possible concentrations), but lengthened in the presence of low-molecular-weight DOACs (dabigatran or rivaroxaban). We explain this by the fact that the rate of direct second-order inhibition reactions is always directly proportional to the concentrations of the target factor, the inhibitor, and the reaction rate constant ( *k*_on_ ) (3) :



It follows that heparins (both nadroparin and UFH) in complex with AT more slowly intercept the first formed molecules of thrombin and FXa than DOACs, since, on the one hand, the rate constants *k*_on_ for the thrombin or FXa association with heparin–AT complexes are approximately 2 to 10 times lower than the corresponding constants for DOACs, 43 45 and on the other hand, the molar concentration of heparins at their therapeutic concentrations is much lower than for DOACs. For nadroparin, taking into account its molecular weight (4,500 kDa) and specific activity (90 anti-Xa IU/mg), 32 this concentration at dose 0.6 anti-Xa IU/mL is 1.5 nM, while for dabigatran and rivaroxaban in prophylactic doses −0.4 μM and 0.3 μM, respectively (see Supplementary Material, section 1.2 [available in the online version only]). We were unable to find the *k*_on_ constant for the binding of dabigatran to thrombin, but similar constants for the binding of some other low-molecular-weight inhibitors (LB30870, melagatran, argatroban) to thrombin are reported in the literature. The values of all these constants range from 0.9 × 10 ^7^ to 1.3 × 10 ^7^ M ^−1^ ·s ^−1^ , 46 while a constant for the binding of the AT–nadroparin complex to thrombin is 5.3 × 10 ^6^ M ^−1^ ·s ^−1^ . 43 For the binding of FXa to rivaroxaban or the AT–nadroparin complex, the corresponding constants are 1.7 × 10 ^7^ M ^−1^ ·s ^−1^ and 1.3 × 10 ^6^ M ^−1^ ·s ^−1^ , respectively. 43 45 Thus, DOACs more quickly intercept the first formed molecules of thrombin and FXa, which lengthens the time to reach their concentrations necessary for the onset of accelerated coagulation, lengthening the lag period of clotting compared to heparins.

The final size of the clot is determined by how far thrombin spreads in space. this depends not only on how much thrombin was formed but also on the anticoagulant used. The main role in the spatial distribution of thrombin is played by FIXa and FXI. FIXa has been shown to be a major factor determining the spatial distribution of coagulation, since unlike thrombin and FXa, it has a long plasma lifetime and can diffuse quite far from its site of formation. 2 14 47 Factor XI is evenly distributed in plasma. Thrombin activates FXI at all points where it appears and can trigger a coagulation cascade at these new points, leading to thrombus growth. Since heparins significantly enhance FIXa inhibition, this may explain the decreased rate of fibrin propagation and the formation of smaller clots in their presence, in contrast to DOACs that do not inhibit FIXa.

Reversible DOACs form inactive complexes with the target factor, which can diffuse in space much further than free factors. However, since there is an equilibrium between inhibitor-bound and unbound forms of the factor, the breakdown of the factor–inhibitor complex can result in a free factor away from the activator (where the factor–DOAC complex reaches during diffusion) and trigger a coagulation cascade. According to our hypothesis, this is the main mechanism influencing the occurrence of recurrent hypercoagulation in a patient with a decrease in the concentration of any DOAC in the blood. The binding of the active factor to the DOAC gradually decreases. There is no longer a free inhibitor in the blood, but for some time the complex factor with DOAC remains in the plasma, and its dissociation can lead to the recurrence of thrombosis. A similar situation cannot be observed in the case of the heparins introduction.

Thus, the clot grows only while thrombin, followed by fibrin, spreads in space, that is, the clot growth rate can determine its size. We assume that this parameter is critical for hemostasis and should be stabilized. This study confirmed that, unlike the TGT parameters, the clot growth rate actually has a decreased coefficient of interindividual variability. 18 48 49 Besides, it can be assumed that most of the thrombin, formed after the appearance of a thrombus, is necessary not only for the activation of platelets, the protein C system, thrombin activatable fibrinolysis inhibitor, etc. but also for maintaining the clot growth rate in space.

A limitation of our study is the relatively small size of the groups studied ex vivo (33, 27, and 15 patients took rivaroxaban, dabigatran etexilate, and nadroparin, respectively), and the small number of anticoagulants explored. Therefore, we currently consider our results as qualitative. However, we also obtained similar results for UFH in vitro (see Supplementary Material, section 2.6 and Supplementary Fig. S2 [available in the online version only]), confirming the fundamental difference between heparins and DOACs.

## Conclusion

Our work was the first to demonstrate a fundamental difference in the mechanisms of action of heparins and DOACs at the stages of activation and spatial propagation of clotting. Simultaneous measurement of parameters characterizing the formation and spatial distribution of thrombin and fibrin became possible due to the TD-4D test.

The main findings of this work, obtained in vitro and confirmed ex vivo, are as follows:

1. Heparins (both UFH and LMWH [nadroparin]) at physiologically possible concentrations have a very weak effect on the activation stage of coagulation, characterized by such parameters as *T*_lag_ and *T*_max_ , and relatively weak on the maximum activity of thrombin formed on the activator ( *C*_max_ ). On the other hand, heparins strongly inhibit the spatial spread of thrombin and fibrin (parameters *V*_t_ , *A*_st_ , and *V*_i_ , *V*_st_ , *V*_f_ , respectively).
2. DOACs, on the contrary, significantly prolong the onset of coagulation ( *T*_lag_ and *T*_max_ ) but have a weaker effect on its spatial distribution than heparins ( *V*_i_ , *V*_st_ , *V*_f_ , as well as *V*_t_ and *A*_st_ ). They also have a moderate effect on *C*_max_ .
3. With a decrease in the concentration of any DOAC (both a thrombin inhibitor and an FXa inhibitor), thromboembolic complications may occur, which are associated with the breakdown of complexes of these reversible inhibitors with their target factors and the release of free active factors far from the activator.

The clinical significance of all these findings is not yet fully clear. Considering that a decrease in the concentration of DOAC may lead to a relapse of the hypercoagulation initially observed in the patient, one can try to discontinue DOAC against the background of a minimum dose of heparin (for 1–2 days) in order to maintain the inhibitory capacity of the patient's own AT and after 2 to 3 days transfer the patient only to LMWH.

Since re-thrombosis is possible due to the breakdown of factor–DOAC complexes from which the factor is released active, one could try to create antibodies or another type of “antidote” that would prevent the breakdown of these complexes. This would make the anticoagulant irreversible and eliminate the cause of coagulation initiation away from the activator. In any case, the clinical application of the presented results requires further in-depth studies.

## Notes

**What is known about this topic?**

- Various reactions are involved in the phases of coagulation activation and propagation of coagulation in space. How different anticoagulants act on these phases has not been studied.
- Various heparins inhibit coagulation effectively but require intravenous or subcutaneous administration and monitoring.
- DOACs have approximately the same pharmacokinetics for most patients, so they can be used in the same doses without monitoring.

**What does this paper add?**

- The formation of a fibrin clot is determined not only by the amount of thrombin formed but also by its ability to spread in space. The same amount of thrombin can form fibrin clots of significantly different sizes depending on the mechanism of thrombin inhibition (AT-dependent or -independent).
- Heparins delay spatial propagation of coagulation, but not coagulation activation since they enhance the factor IXa inhibition by AT.
- Dabigatran and rivaroxaban inhibit the activation of clotting more than its spatial spread, since they do not inhibit FIXa, but intercept the first-formed thrombin and FXa molecules faster than AT–heparin complexes, which prolong the period of clotting delay.
