# Supplementary material for: Heparin and Direct Oral Anticoagulants have Different Effects on the Phases of Activation and Spatial Spread of Blood Coagulation
Source: Thromb Haemost. 2025 Feb 17;126(1):62–77. doi: 10.1055/a-2516-7384 (PMC12758956; doi:10.1055/a-2516-7384)
Supplement: Supplementary file 1 — Supplementary Material [file 10-1055-a-2516-7384-s24110581.pdf]

Heparin and direct oral anticoagulants have different effects on the phases of activation and spatial spread of blood coagulation

Fazoil I. Ataullakhanov, Natalya M. Dashkevich, Ruzanna A. Ovsepyan, Tatiana A. Vuimo, Anna N. Balandina, Anna D. Kuprash, Dorzo-Cyren B. Ayusheev, Alexey I. Bernakevich, and Elena I. Sinauridze

SUPPLEMENTARY MATERIAL

1. Used anticoagulants

Dabigatran and rivaroxaban (Selleckchem, Houston, TX, USA) stock solutions for *in vitro* work were prepared in dimethylsulfoxide and stored at -80 °C. Unfractionated heparin (UFH) was produced by Bryntsalov-A (Moscow, Russia) and nadroparin calcium (Fraxiparine) was produced by Aspen Pharma Trading Limited (Dublin, Ireland). Dabigatran etexilate (Pradaxa) was from Boehringer Ingelheim Pharmaceuticals, Inc. (Ingelheim am Rein, Germany), and rivaroxaban for clinical use (Xarelto) from Bayer HealthCare Pharmaceuticals (Berlin, Germany).

1.1. Doses of anticoagulants used for thromboprophylaxis

The following prophylactic doses of anticoagulants were used *ex vivo*: dabigatran - 220 mg once a day;[1] rivaroxaban - 10 mg once a day[2] and nadroparin in a total dose of 5700-8550 IU anti-Xa per day (for two injections) depending on the patient's weight (less than or more than 90 kg, respectively). This corresponds to a maximum dose of approximately 95 IU anti-Xa/kg and can result in a Amax of approximately 0.58 IU anti-Xa/ml.[3] A dose of 6500 IU anti-Xa nadroparin (twice daily) is called a high prophylactic dose.[4] In accordance with this literature, the effect of these prophylactic doses is approximately similar to the effect of standard dose enoxaparin (4000 IU anti-Xa/day). The range of anticoagulant concentrations in *in vitro* experiments was wider and included both prophylactic and therapeutic doses.

To compare *in vitro* and *ex vivo* experiments, anticoagulant concentrations corresponding to those obtained *ex vivo* were used (see below).

1.2. Calculation Amax for studied anticoagulants after administration of their prophylactic doses *in vivo*

Patients with a minimum weight of 60 kg (to <90 kg) received a total dose of nadroparin of 5700 IU, and patients with a minimum weight of 90 kg and above received a total dose of 8550 IU. Thus, in both cases, the maximum dose of nadroparin was 95 IU/kg. According to,[3] after converting nadroparin units from IC U to IU (1 IC U = 0.413 IU), administration of a nadroparin dose of 41.3 IU/kg results in Amax of 0.252 IU/kg. Given that for LMWH the dose of the drug is directly proportional to its maximum plasma concentration, we obtain that a dose of 95 IU/kg should result in Amax = 0.58 IU/mL, i.e. approximately Amax = 0.6 IU/mL.

Following a dose of dabigatran etexilate 220 mg once daily, the Amax for dabigatran can be calculated according to Stangier J,[5] and is approximately 186.4 µg/mL or 0.4 µM.

Amax after a single administration of 10 mg rivaroxaban to patients was measured in works [6] and [7] (0.28 µM and 0.29 µM, respectively). Thus, we took it as 0.3 µM.

1.3. Maximum possible plasma concentrations of the studied anticoagulants after the administration of their therapeutic doses

The limits of the maximum possible plasma concentrations of various studied anticoagulants in Figures 3 and 4 (main text) are indicated conditionally according to literature data. Thus, for nadroparin it is known that the maximum concentration in plasma after subcutaneous administration can reach 1.55 anti-Xa IU/mL.[8] The maximum clinical dose of dabigatran is 300 mg/day. As a result, the maximum concentration of dabigatran in plasma can reach approximately 450-600 μg/L with the administration of 150 mg x 2 times a day for 7 days,[9] which corresponds to approximately 1.11 mM. A similar maximum plasma concentration of rivaroxaban can be estimated as 500 ng/ml (i.e., an average of approximately 1.15 μM).[7] The concentration of UFH in plasma (see Fig. S2 below) during therapy can reach 0.7 anti-Xa IU/mL.[10]

2. Thrombodynamics-4D test

2.1. Device and reagents for measurements

Commercially available Thrombodynamics® Analyzer T2T and reagent kits for Thrombodynamics-4D assay were provided by HemaCore LLC (Moscow, Russia). The kits included cast plastic chambers (HemaCore S.A., Monthey, Switzerland) and activators covered with immobilized tissue factor (TF) with a surface density of 100 pmol/m^2. Kits contained also the following lyophilic-dried reagents: contact activation inhibitor (corn trypsin inhibitor) together with thrombin-specific fluorogenic substrate (Z-Gly-Gly-Arg-AMC, where AMC is 7-amino-4-methylcoumarin), PLS-reagent (a mixture of phospholipid vesicles containing phosphatidylserine and phosphatidylcholine in molar ratio of 1:4, respectively), and calcium acetate reagent.

2.2. Blood collection and plasma preparation

Blood for analysis was collected from the ulnar veins of volunteers or patients into standard plastic vacuum tubes with 3.2% (0.109 M) trisodium citrate dihydrate solution (Vacuette tubes, Greiner Bio-One GmbH, Kremsmunster, Austria). The volume ratio of blood to citrate was 9:1. The first tube after the venipuncture was discarded. Platelet-free plasma (PFP) was immediately obtained by two sequential blood centrifugations: 15 min at 1,600 g and 5 min at 10,000 g at room temperature. Part of the obtained PFP samples was then frozen in liquid nitrogen and stored at -80 ^{o}C.

2.3. Measurement and parameters

First, the PLS reagent was prepared. For this purpose, 300 μL of distilled water was injected into a tube containing lyophilisate of vesicles (20% phosphatidylserine and 80% phosphatidylcholine in a buffer consisting of NaCl, 4-(2-hydroxyethyl)-1-piperazineethanesulfonic acid (HEPES), bovine serum albumin and maltose). The contents were dissolved for 5 min, stirring smoothly.

In a tube with a lyophilized contact activation inhibitor (corn trypsin inhibitor) and thrombin-specific fluorogenic substrate (Z-Gly-Gly-Arg-AMC), 120 μL of platelet-free plasma and 5 μL of the prepared liquid PLS reagent were added. In *in vitro* experiments, various concentrations of the

studied anticoagulants were preliminarily added to plasma (2 μL of anticoagulant solution per 118 μL of plasma). The mixture was incubated for 3 min at 37 °C.

The incubated mixture (120 μL) was then transferred to a tube with a lyophilized calcium acetate reactant. The contents were quickly dissolved and placed in a cuvette for measurement. The plate, the end of which was covered with an immobilized activator (TF), was carefully inserted into the cuvette from above.

Simultaneous measurement of light scattering (at λ=625 nm) and fluorescence of the AMC (at $\lambda_{excitation}$ = 365 nm; $\lambda_{emission}$ = 440 nm) was performed. The results were recorded using a CCD camera every 6 seconds. The obtained results were processed using an automated calculation algorithm by software specially developed by manufacturers.

The fibrin occurrence was characterized by the light scattering. The clot growth rate was calculated from the movement of the point of half-maximal intensity of the light scattering profiles. AMC fluorescence at different distances from the activator gave the spatial distribution of thrombin at different times.

Fibrin formation is determined by the following parameters:

Tlag is the time between the coagulation activation upon contact with TF and the onset of fibrin formation on the activating surface.

The curves of clot size *vs*. time were constructed to measure the initial (Vi) and stationary (Vst) clot growth rates (using the slopes of these curves at segments 2–6 min and 15–25 min from the beginning of clot growth for Vi and Vst, respectively).

Vf (or $Vf_{45\text{-}55\ min}$) is the rate of clot growth in the time interval 45-55 min after the coagulation activation. It shows how a fibrin clot propagates in space far from the activator, where the influence of TF is minimal.

Thrombin generation at the activating surface was characterized by the height of the thrombin peak near the activator (Cmax) and the time to reach this peak (Tmax).

The summarized thrombin generation near the activator in the range of 0.05-0.2 mm (TG_A) was also calculated for all the time of experiment. This indicator is not the same, but is close to the endogenous thrombin potential (ETP), usually measured in the standard homogeneous test of thrombin generation (see below).

Vt (or $Vt_{45\text{-}55\ min}$) is the rate of thrombin propagation in the time interval of 45-55 min after activation. This rate is measured as the rate of AMC propagation at a point at half the height of the front edge of the thrombin distribution.

Vf and Vt characterize the processes of fibrin and thrombin propagation over the same time interval (45-55 min after activation). At this time, thrombin and fibrin spread far from the activator where its influence is minimal. These rates were shown to vary between samples, but were similar within each sample with good accuracy (Fig. S1).

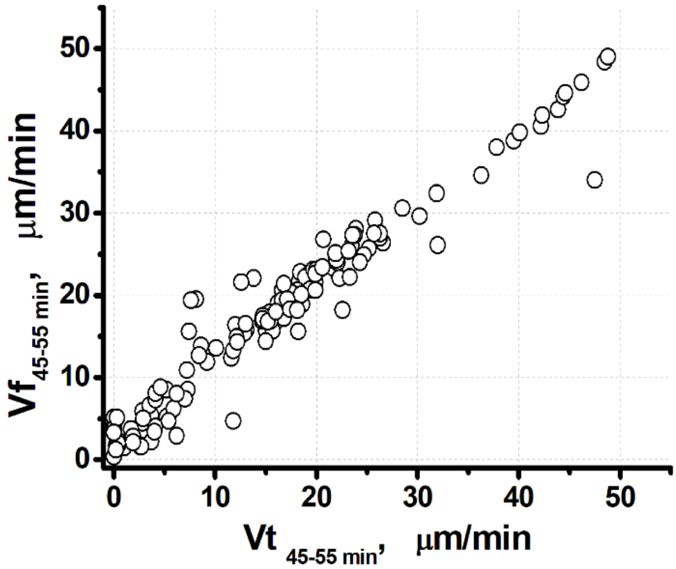

Figure S1. Correlation of spatial rates of thrombin ($Vt_{45\text{-}55\ min}$) and fibrin ($Vf_{45\text{-}55\ min}$) propagation over a time interval of 45-55 min after activation (according to all data from *in vitro* experiments).

Another important parameter is the time when spontaneous clots, which are not associated with the activator surface, appear in the sample, (Tsp, min). The presence of such clots indicates plasma hypercoagulation.[11] To determine this time, the light scattering signal is continuously summed up over a certain part of the cuvette area, sufficiently distant from the activator. By definition, the time of spontaneous clots formation is taken to be the time when the plasma light scattering intensity in this zone reaches 5% of its possible maximum intensity (see Fig. S6**D** below).

2.4. Measurement of thrombin generation by Thrombodynamics-4D

Due to the characteristics of the optical system of the Thrombodynamics® Analyzer T2T and the image processing algorithms, the spatial distribution of thrombin cannot be reliably measured at a distance of less than 0.2 mm from the activator, but the average thrombin activity in this area can still be calculated. To characterize the initial phase of thrombin generation, which occurs directly at the TF-coated activator's surface, and to calculate the thrombin activity in this area, we used the average AMC concentration in the range of 0.05-0.2 mm from the activator. It should be noted, that we did not measure the thrombin concentration (in nM), but the fluorescence of the formed thrombin in arbitrary units of fluorescence per liter (A.U./L), and in all the figures we compared these values. The A.U./L value can be recalculated in thrombin concentration (in nM) if the calibration signal of the exact AMC concentration is measured in a separate sample of the plasma being investigated. Since the magnitude of the AMC calibration signal may vary between plasma samples by about ± 6%, [12] comparison of thrombin concentrations determined in A.U./L is likely to result in increased variations (errors) in the measured thrombin generation parameters compared to the fibrin propagation parameters. This does not affect the results at a work with the same plasma, but may increase variations between the plasmas of different patients. In this work, increased relative errors (CVerr) were indeed observed when measuring thrombin generation parameters compared to measuring clot growth parameters.

If necessary, the Thrombodynamics® T2T Analyzer allows you to measure the ETP in a standard performance. In this case, the soluble clotting activator must be placed in a sample and immediately mixed, after which, as in the standard TGT, the profiles of AMC formed as a result of the thrombin reaction with a fluorogenic substrate are recorded. The amount of active thrombin formed in the sample during the entire experiment represents the standard ETP.

2.5. Normal values, reproducibility and inter-individual variability of TD-4D parameters

To evaluate the reproducibility of TD measurements, we performed 14 series of measurements in pooled plasma from 3 donors. Each series was performed as N=7-14 simultaneous measurements of the same plasma sample by one operator using one lot of reagents (total number of repeated measurements N = 146). Two measurements can be performed simultaneously using one Thrombodynamcs® T2T Analyzer, therefore 4-7 instruments were used together. For each series, the coefficient of variation of each parameter, characterizing the relative measurement error (CVerr), was estimated. The reproducibility of each parameter was characterized by its average CVerr calculated from all series of experiments taking into account the share of each series in the total pool of measurements. All CVerr were calculated according to equation (1), since CVerr distributions were normal for all parameters.

$$CVerr=SD\times 100/Mean \qquad (1),$$

where SD is the standard deviation, and Mean is the mean value of the parameter, averaged for all repeated experiments conducted in similar conditions.

Interindividual coefficients of variation (CVii) for each parameter in the group of healthy donors (N=21) were calculated in accordance with the study of Sinauridze EI, et al.[13] using equation (2):

$$CVii = (CVtotal^2 – CVerr^2)^{1/2} \qquad (2),$$

where CVtotal is the total coefficient of variation for each group parameter, equal to SD×100/Mean, where SD and Mean are calculated for the study group.

The minimum CVerr were obtained for the initial and stationary clot growth rates Vi and Vst (CVerr 3.45% and 3.60%, respectively). The relative errors were higher, for thrombin generation parameters, as well as for Tlag and Tmax measurements (if we assume that these parameters are also distributed normally). The highest value of CVerr was obtained for the height of the mobile peak of thrombin at 60 min (Ast) (CVerr 13.84%). The mean values of the main parameters of the TD-4D assay in normal plasma were measured previously in the work of Koltsova EM, et al.[14] Here we repeated such measurements for healthy donors, whose samples we used to measure interindividual variability of different parameters of the TD-4D test. The obtained values were quite close to those previously published (Table S1).

The lowest interindividual variability (3.32%) was observed for the initial clot growth rate (Vi), while Ast and TG_A had the highest interindividual variability (25.70% and 27.34%, respectively). The parameters Vst, Vt, as well as Cmax had CVii equal to 4.34%, 14.03% and 24.14%, respectively. Thus, all parameters characterizing the thrombin generation have higher coefficients of interindividual variations in the population compared to the parameters of fibrin clot propagation. As mentioned above, this situation may be partly due to the fact that instead of

the exact concentration of thrombin, a fluorescence value (without calibration) was used for the calculation. The mean ± SD was used as the normal range for TG_A, and the mean ± 2 × SD for all other TD-4D parameters (except the non-normally distributed Tlag and Tmax). Ranges including 2.5-97.5 percentiles of all values were accepted as normal ranges for these non-normally distributed parameters.

Table S1. Mean values of the Thrombodynamics-4D parameters, relative errors of their measuements (CVerr) and their interindividual variability (CVii) in the plasma of healthy donors[a)].

Parameter, units	Mean (or median)	SD (or range)	CVtotal, %	CVerr, %	CVii, %
Tlag, min	(0.8)	(0.3-1.3)	-	14.43	-
Vi, µm/min	66.09	3.16	4.79	3.45	3.32
Vst, µm/min	37.96	2.14	5.64	3.60	4.34
Cmax, A.U./L	415.23	109.08	26.27	10.36	24.14
Ast, A.U./L	71.89	20.98	29.19	13.84	25.70
Vt, µm/min	30.74	5.70	18.55	12.13	14.03
Tmax, min	(1.6)	(1.5-2.1)	-	10.67	-
TG_A, (A.U./L)×min	2022.3	569.0	28.14	6.65	27.34

a) All the parameters excluding Tlag and Tmax have normal distribution. The following parameters for a group of healthy donors (N=21) are presented: mean values (or median for Tlag and Tmax), standard deviation SD (or range 2.5-97.5% for Tlag and Tmax), relative error of each parameter measurements (CVerr), total (CVtotal) and interindividual (Cii) coefficients of variation. CVtotal and CVii for Tlag and Tmax were not calculated, since the values of these parameters were not normally distributed.

2.6. Effects of UFH on TD-4D parameters *in vitro*

The effect of UFH on TD-4D parameters was studied *in vitro*, similar to LMWH (nadraporin) (Fig. S2).

3. The basic characteristics of the patients in subgroups, obtaining different anticoagulants

The general characteristics of patients in each group (receiving nadroparin, dabigatran etexilate or rivaroxaban), as well as the number of asymptomatic thrombosis of the lower extremities on 7-8 days of therapy in each group are presented in Table S2.

The study included all patients undergoing knee or hip replacement surgery at the National Medical Research Center for Traumatology and Orthopedics named after N.N. Priorov (CITO, Moscow, Russia) from February 2015 to June 2016 who did not meet the exclusion criteria: 1) age less than 18 years; 2) presence of hematological diseases; 3) taking anticoagulants within 2 weeks

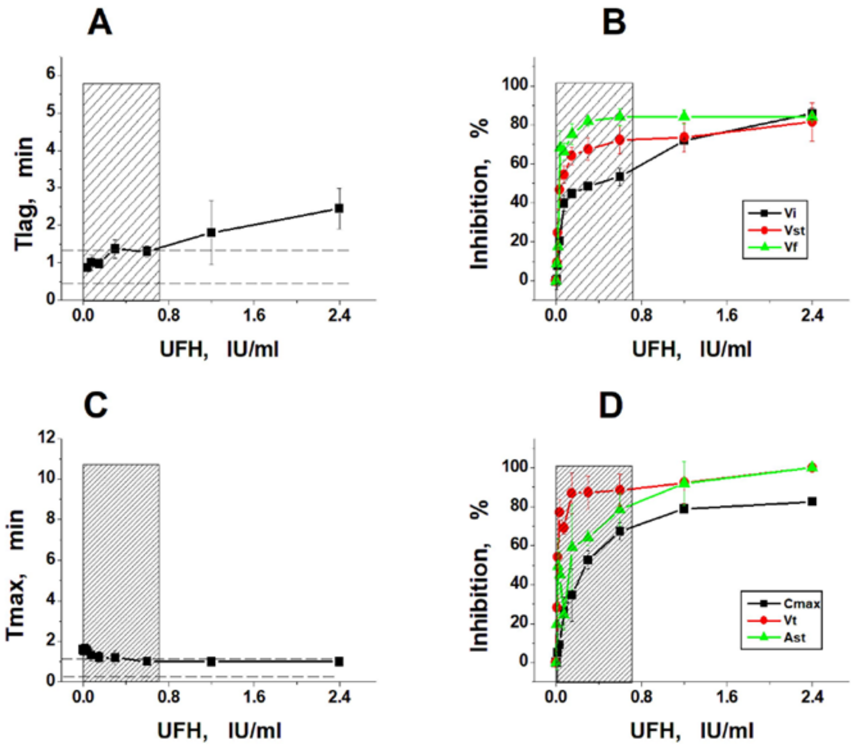

Figure S2. Effects of UFH *in vitro* on TD-4D parameters characterizing fibrin formation: Tlag, (**A**), Vi, Vst, and Vf (**B**), as well as parameters characterizing thrombin generation and propagation in space: Tmax (**C**), Cmax, Vf, and Ast (**D**). The mean values ± SDs are presented. N=5. The shaded areas represent the plasma concentrations that can be obtained during therapeutic or prophylactic treatment (see SM, section 1.3). The horizontal dashed lines represent the normal ranges for the Tlag and Tmax values.

Table S2. The basic characteristics of patients in different subgroups.

**Subgroup**	**N total**	**Gender F/M**[a)]	**Age, years (mean±SD)**	**Weight, kg (mean±SD)**	**Type of surgery, (hip/knee)**[b)]	**Number of asympto-matic thrombosis on 7-8 days**[c)]	**Thrombosis localization**
Nadroparin	15	10/5=2.0	62.4 ± 14.8	81.8+16.3	11/4	0/10 (0%)[d)]	-
Dabigatran etexilate	27	17/10=1.7	59.1±13.0	79.2.0+20.5	20/7	3/27 (11%)	Thrombosis of the gastrocnemius muscle -1. Ultrasound signs of occlusive distal thrombosis of v. Soleus, at the level of the middle third - 2.

Rivaroxaban	33	23/10=2.3	57.5±12.9	79.0+14.4	22/11	6/21 (29%)[d)]	Ultrasound signs of occlusive distal thrombosis of v. Soleus, mainly at the level of the upper and middle third - 6.

**Subgroup**	**Concomitant diseases (and the number of such patients)**	**Average volume of transfused plasma substituting solutions (median and range), mL[e)]**
Nadroparin	CAD – 5, cardiac insufficiency – 1, AH 1 – 1, AH 2 – 6, obesity stage I, diabetes mellitus type II – 2, rheumatoid arthritis -1, chronic cholecystitis – 2, chronic gastritis – 2, chronic gastroduodunitis – 1, varicose veins – 3, chronic venous insufficiency – 1, thyroid nodules – 1, uthyroidism – 1, left kidney cyst – 2, biliary reflux – 1, impaired glucose tolerance – 1, chronic pyelonephritis – 1, kidney stone disease – 2, micronephrolithiasis – 1, nephroptosis – 1, hemangioma of the live - 1	3170 (2252-7200)
Dabigatran etexilate	CAD – 6, AH 1 – 3, AH 2 – 11, obesity (degree 1) – 2, obesity (degree 2) – 4, gastritis – 6, varicose veins – 6, chronic venous insufficiency – 2, rheumatoid arthritis – 1, nodular goiter – 1, pulmonary sarcoidosis – 1, microliths and cysts of both kidneys – 1, duodenitis – 1, hypothyroidism – 3, hyperparathyroidism – 1, chronic bronchitis – 1, gout – 1, chronic cholecystitis in remission – 2, fatty hepatosis – 1, kidney stone disease – 2, chronic kidney disease – 1, diabetes mellitus (type 2) – 1, cholestasis – 1, atherosclerotic cardiosclerosis – 1, pneumosclerosis – 1, cancer of the breast T2N0M0, (radical mastectomy 2.5 years ago) – 1, microliths and cysts of both kidneys – 1, chronic pancreatitis – 1.	3400 (1000-7390)
Rivaroxaban	CAD -2, AH 2 – 11, AH 3 – 1, heart arrhythmia – 3, Cardiac insufficiency – 3, obesity (degree 1) – 2, obesity (degree 2) – 3, gastritis – 10, gastric polyp – 1, duodenal ulcer (in remission) – 3, chronic gastroduodenitis – 7, duodenogastric reflux – 1, chronic reflux esophagitis -1, duodenal dyskinesia – 1, varicose veins – 4, chronic venous insufficiency – 4, phlebectomy – 1, juvenile rheumatoid polyarthritis – 1, kidney cysts – 4, cholecystectomy – 1, subclinical hypothyroidism, – 1, gout – 1, micronephrolithiasis – 1, kidney stone disease – 1, chronic pyelonephritis (in remission) – 1, diabetes mellitus (type 2) – 3, rectal cancer T3N0M0 (metastasis to the left lung, resection of the lower lobe of the left lung 8 months ago) – 1, chronic cholecystitis in remission – 3, hepatitis – 1, fatty hepatosis – 3, atherosclerotic cardiosclerosis – 1, pneumosclerosis – 1, pulmonary emphysema – 1, fibrosis and cirrhosis of the liver (in compensation) – 1, acute cerebrovascular accident 20 years ago – 1, hiatal hernia – 1.	4050 (3000-6200)

[a)] F – female, M – male; [b)] the number of operations on the hip or knee joints is indicated; [c)] duplex scanning of the lower extremities veins was performed to determine asymptomatic thrombosis; [d)] the ultrasound investigation was performed only in 10 and 21 patients in the nadroparin and rivaroxaban groups, respectively, the ratio of the number of thromboses to the total number of measurements is indicated; [e)] the average total volume of plasma-substituting solutions transfused to patients during and after surgery is presented. CAD - coronary arteries disease (ischemic heart disease); AH 1 - AH 3 - arterial hypertension (degrees 1, 2 or 3).

before hospitalization; 4) pregnancy; 5) refusal to participate in the study. No selected patients were withdrawn from the study during treatment. All 3 groups did not differ significantly in terms of the ratio of patients of different sex, the average age and average weight of patients in each group (one way two-tailed ANOVA, *P*<0.05).

4. Diagram of measurement points in ex vivo experiments

A diagram of measuring the parameters of TD-4D in patients indicating the moments of anticoagulant administration, as well as measurement points corresponding to its maximum and minimum concentration, is shown in Fig. S3 (on the example of the stationary clot growth rate (Vst) in the group of patients treated with nadroparin). Point 1 corresponds to a measurement 1 h before surgery (Day 1). The points in the red rings correspond to the moment without anticoagulant (Day 2, before the first anticoagulant administration) and other moments when there is a minimum dose of the anticoagulant (at the end of the action of the preparation dose, prior to the next anticoagulant administration). Arrows show the moments of anticoagulant administration, and horizontal brackets connect adjacent points with maximum and minimum anticoagulant concentrations (2/3, 4/3, 4/5 and 7/6). For these pairs of points, the significance of the differences in the parameter values was calculated using the Wilcoxone T-test with Holm's correction for multiple comparisons (the difference was considered significant at *P*<0.05). The shaded area presents the normal range for the measurement parameter (Vst). Similar diagrams are correct for all parameters of any of the studied anticoagulants.

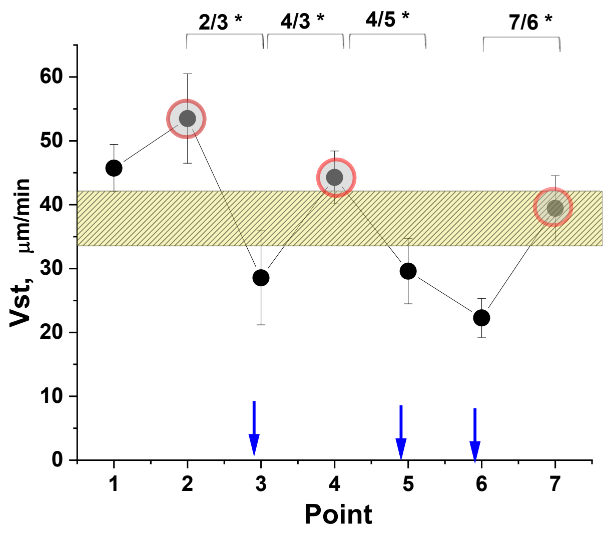

Figure S3. Typical scheme of study points (on the example of Vst in the group of patients treated with nadroparin (N=15)). Points 3, 5 and 6 correspond to the maximal action of nadroparin (3 h after its dose administration). At point 2 nadroparin was not yet administered. Points 4 and 7 correspond to the end of the nadroparin dose action (12 h after administration at points 3 and 6, respectively). The significance of differences was calculated for the following pairs of points: 2-3, 4-3, 4-5 and 7-6, using g the Wilcoxon test with Holm's correction for multiple comparisons. Shaded area corresponds to the normal range of the parameter. Blue arrows indicate the moments of nadroparin administration. Mean values ± SEM are presented. This scheme is applicable for all studied anticoagulants. * - Significant difference.

5. Clot growth and thrombin generation at high concentrations of anticoagulants

Therapeutic plasma concentrations of nadroparin can reach 1.55 IU mL, and dabigatran and rivaroxaban – 1.11 and 1.15 μM, respectively (see earlier section 1.3).

Interestingly, at high plasma concentrations well above therapeutic concentrations, the anticoagulants studied behaved quite differently (Fig. S4). At a plasma LMWH nadroparin concentration of 4.8 anti-Xa IU/mL, the maximum thrombin concentration in plasma was greatly reduced, and the rate of its propagation in space and, accordingly, the clot growth rate decreased to almost 0 (Fig. S4**A**, **B**). For oral anticoagulants, thrombin production also decreased, but the clot growth rate did not decrease so significantly (Fig. S4**C**-**F**). The clot continued to grow even when the sample with dabigatran (9.6 μM) had very low levels of thrombin (Fig. 4S**C**, **D**). A similar effect was observed also for rivaroxaban (9.6 μM) (Fig. S4**E**, **F**).

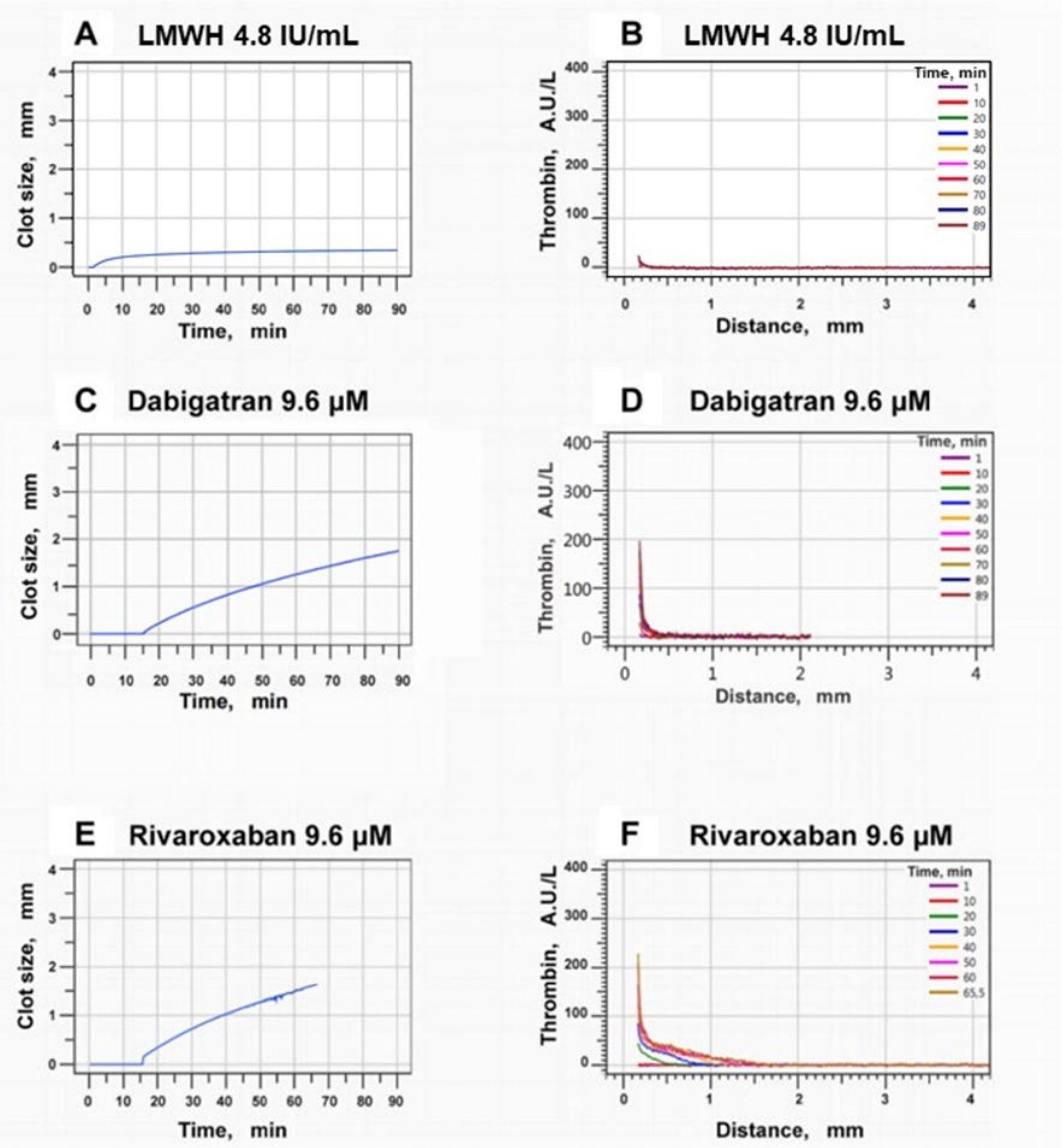

Figure S4. Typical examples of clot growth (**A**, **C**, **E**), as well as thrombin generation (**B**, **D**, **F**) dependences *vs*. time in plasma *in vitro* at high concentrations of different anticoagulants. **A**, **B** – LMWH nadroparin (4.8 anti-Xa IU/mL); **C**, **D** – dabigatran (9.6 μM); **E**, **F** – rivaroxaban (9.6 μM). The times corresponding to all curves are determined in panels **B**, **D** and **F**.

Although dabigatran directly inhibits thrombin, reducing its plasma levels to such low values that they cannot be measured automatically by the Thrombodynamics® Analyzer T2T, clot growth continued even at dabigatran concentrations as high as 20 µM. Typical examples of the spatial thrombin distribution, clot light scattering profiles, and the dependence of clot size *vs*. time in the plasma of one of the donors for two dabigatran concentrations (5 µM and 20 µM) are presented in Fig. S5.

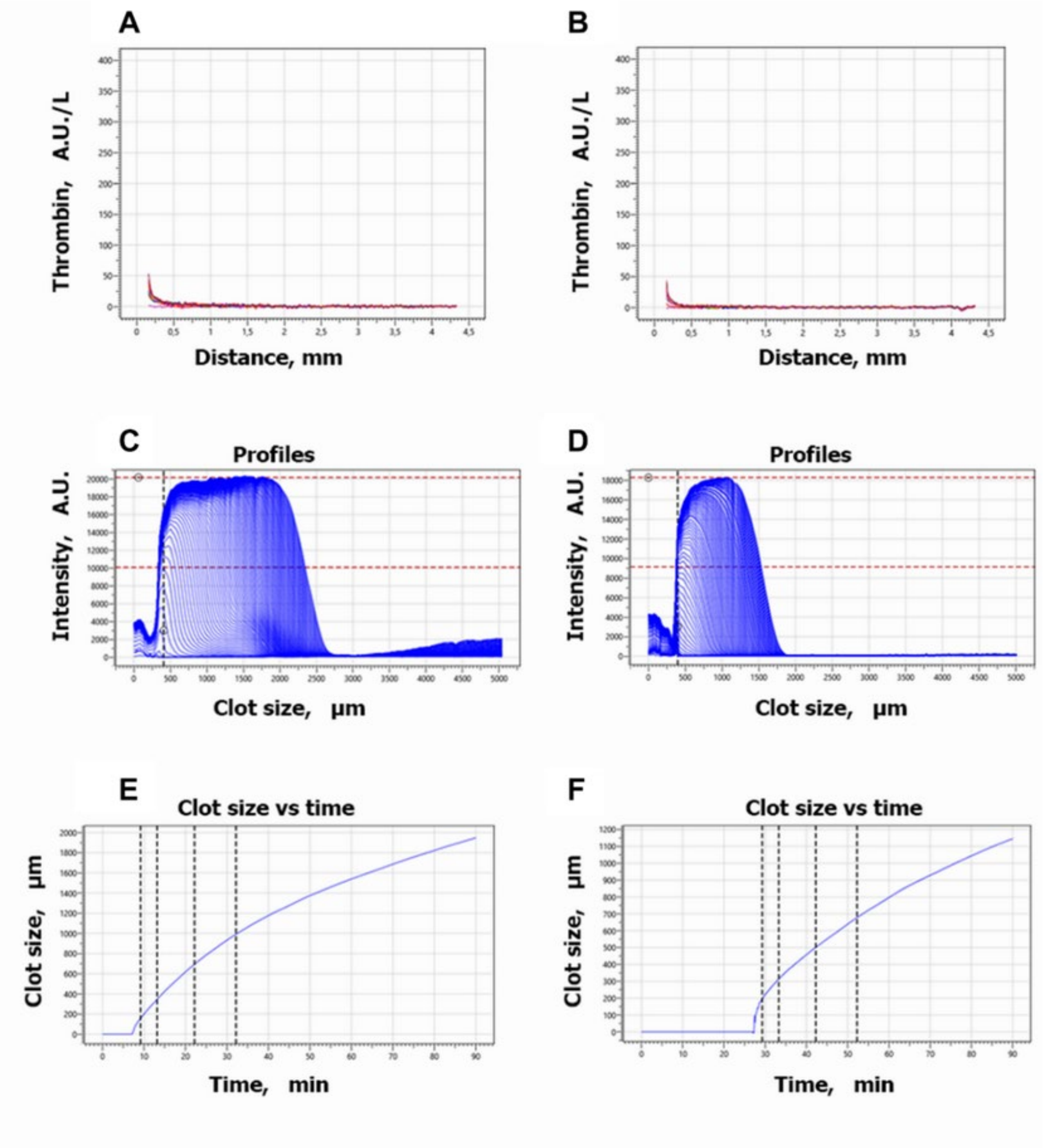

Figure S5. Parameters of clot growth and thrombin formation at high plasma concentrations of dabigatran in blood plasma of one of the donors (*in vitro*). (**A**, **C** and **E**) – The dabigatran concentration is 5 µM; (**B**, **D** and **F**) – the dabigatran concentration is 20 µM. (**A**, **B**) – Spatial distribution of thrombin at 1, 30, 60 and 90 min. (**C**, **D**) – Light scattering profiles of clots at different times after coagulation activation. Horizontal lines indicate the maximum value of light scattering and the value corresponding to half of this maximum value. Vertical lines mark the beginning of reliable measurements (a level where there is no distortion of profiles because of the optical properties of the measuring system). (**E**, **F)** – Kinetics of clot growth (dependence of clot size *vs.* time). Vertical lines mark time intervals for calculation of Vi and Vst.

The values of the parameters calculated using this data are as follows:

Dabigatran 5 µM

Tlag = 7.1 min
Vi = 48.5 µm/min
Vst = 26.7 µm/min

Dabigatran 20 µM

Tlag = 27.7 min
Vi = 23.1 µm/min
Vst = 17.7 µm/min.

Thrombin concentrations were calculated manually because they were low and could not be calculated automatically.

6. Significance of differences between pairs of adjacent points with minimal and maximal drug effect in patients treated with various anticoagulants

Paired Wilcoxon T-test (Wilcoxon signed-rank test) was used for calculations together with the Holm correction for multiple comparisons, which was performed using the "stats" package in R (v 4.4.0). Differences were considered significant at *P* values <0.05.

The following parameters were investigated: Tlag, Vi, Vst, Cmax, Vt, and Ast. A comparison was performed between pairs of points indicated in Fig. S3. Dabigatran etexilate (Pradaxa) and rivaroxaban (Xarelro) were administered once a day, nadroparin (Fraxiparine) – 2 time a day (all the measurements in this case were carried out in the morning (before or after the first dose of nadroparin on the corresponding day). Point 2 is a sample after operation (without anticoagulant). Points 3, 5 and 6 correspond to the time, when the action of the anticoagulant dose is maximal, points 4 and 7 correspond to the final moment of the anticoagulant dose action (24 h after taking of dabigatran etexilate and rivaroxaban, or 12 h after nadroparin injection).

P values for significant (*P*<0.05) and insignificant (*P*>0.05) differences are marked in the tables in green and yellow, respectively.

Table S3. Significance of differences between different studied pairs of points (at minimum and maximum drug effect) in patients treated with LMWH nadroparin, dabigatran etexilate or rivaroxaban.

Parameter	Pair	N (pairs)[a)]	P- value (paired Wilcoxone T-test)	Adjusted P-value after Holm's amendment
**Nadroparin**				
Tlag	2-3	12	0,09916	0,29749
	4-3	14	0,14099	0,29749
	4-5	14	0,01732	0,06928
	7-6	2	1,00000	1,00000
Vi	2-3	12	0,03098	0,06197

	4-3	14	0,00110	0,00439
	4-5	14	0,00317	0,00952
	7-6	2	0,37109	0,37109
Vst	2-3	9	0,04401	0,13203
	4-3	11	0,01128	0,04511
	4-5	11	0,06835	0,13671
	7-6	2	0,37109	0,37109
Ast	2-3	4	0,20124	0,60373
	4-3	8	0,04232	0,16926
	4-5	9	0,47720	0,74219
	7-6	2	0,37109	0,74219
Vt	2-3	4	0,36131	0,72262
	4-3	9	0,05802	0,23210
	4-5	9	0,23614	0,70841
	7-6	2	0,37109	0,72262
Cmax	2-3	12	0,90633	0,94552
	4-3	14	0,02801	0,11203
	4-5	14	0,31517	0,94552
	7-6	2	0,37109	0,94552
Tmax	2-3	12	0,77283	1,00000
	4-3	14	1,00000	1,00000
	4-5	14	0,48402	1,00000
	7-6	2	1,00000	1,00000
**Dabigatran etexilate**				
Tlag	2-3	20	0,00007	0,00028
	4-3	21	0,60558	0,60558
	4-5	20	0,00419	0,01256
	7-6	20	0,00832	0,01663
Vi	2-3	20	0,00011	0,00045
	4-3	21	0,02178	0,06534
	4-5	20	0,03648	0,07296
	7-6	20	0,53790	0,53790
Vst	2-3	9	0,01285	0,05140
	4-3	18	0,81067	0,81228

	4-5	19	0,30481	0,81228
	7-6	20	0,27076	0,81228
Ast	2-3	3	0,42268	1,00000
	4-3	11	0,75566	1,00000
	4-5	10	0,47553	1,00000
	7-6	18	0,79386	1,00000
Vt	2-3	3	0,42268	1,00000
	4-3	12	0,72408	1,00000
	4-5	12	0,78365	1,00000
	7-6	18	0,51354	1,00000
Cmax	2-3	20	0,04189	0,08378
	4-3	21	0,04757	0,08378
	4-5	20	0,00183	0,00548
	7-6	20	0,00095	0,00381
Tmax	2-3	20	0,00024	0,00097
	4-3	21	0,49112	0,49112
	4-5	20	0,00074	0,00222
	7-6	20	0,00348	0,00697
**Rivaroxaban**				
Tlag	2-3	26	0,00017	0,00067
	4-3	26	0,42519	0,42519
	4-5	29	0,00113	0,00227
	7-6	29	0,000179	0,00067
Vi	2-3	26	0,00002	0,00010
	4-3	26	0,13398	0,13398
	4-5	29	0,00687	0,01374
	7-6	29	0,00071	0,00214
Vst	2-3	17	0,00245	0,00978
	4-3	25	0,91429	0,91429
	4-5	28	0,00319	0,00978
	7-6	28	0,00771	0,01543
Ast	2-3	4	0,36131	0,36131
	4-3	12	0,10780	0,21560
	4-5	19	0,00376	0,01503

	7-6	27	0,02468	0,07405
Vt	2-3	5	0,58964	1,00000
	4-3	14	0,94994	1,00000
	4-5	21	0,04757	0,14271
	7-6	27	0,03249	0,12994
Cmax	2-3	26	0,00088	0,00351
	4-3	26	0,23259	0,23259
	4-5	29	0,001593	0,00478
	7-6	29	0,00327	0,00655
Tmax	2-3	26	0,00003	0,00013
	4-3	26	0,78859	0,78859
	4-5	29	0,00160	0,00319
	7-6	29	0,00014	0,00042

a) Comparisons performed with N<5 pairs are marked in pink.

7. Appearance of spontaneous clots in the plasma of patients receiving different anticoagulants

Figure S6**A**, **B**, **C** shows the percentage of samples containing spontaneous clots after 90 min of measurement at each study point for all anticoagulants examined. The amounts of spontaneous clots decreased after each administration of any anticoagulant. The red arrows indicate the moments of the study when the anticoagulant was administered. Panel **D** shows the principle of calculating the Vi and Vst rates, as well as the times Tsp. The time of spontaneous clots appearance is considered to be the moment when in a certain selected area of the sample, which is far from the activator, the light scattering becomes more than 5% of the maximum possible light scattering in the given plasma.

Figure S7 shows typical examples of spontaneous clots actually observed in plasma samples. Specific examples of light scattering profiles (**A**, **C**, **D**) and clot photographs (at 10, 45 and 90 min after the start of the experiment) from corresponding plasma samples of one of the patients, before surgery (hip replacement) (**B**), after surgery but before taking the anticoagulant (**D**) and 3 hours after taking rivaroxaban (10 mg) (**F**) are shown.

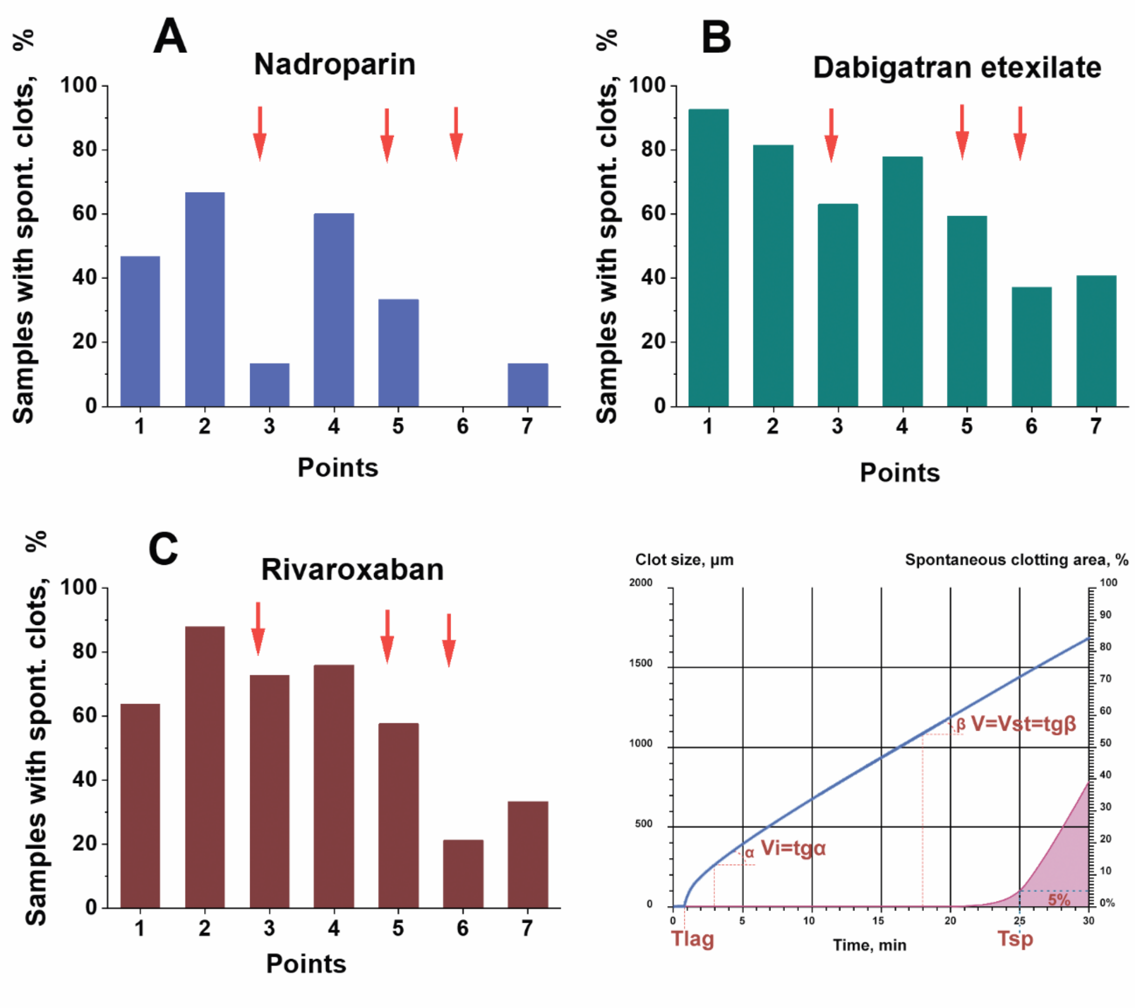

Figure S6. Appearance of spontaneous clots at different points of the study in patients receiving different anticoagulants (percentage of samples with spontaneous clots at the end of measurement shown). **A** - LMWH nadroparin (N=15). **B** – Dabigatran etexilate (N=27). **C** –Rivaroxaban (N=33). Arrows indicate the moments of anticoagulant administration. **D** – principles of calculating the parameters Vi and Vst, as well as Tsp.

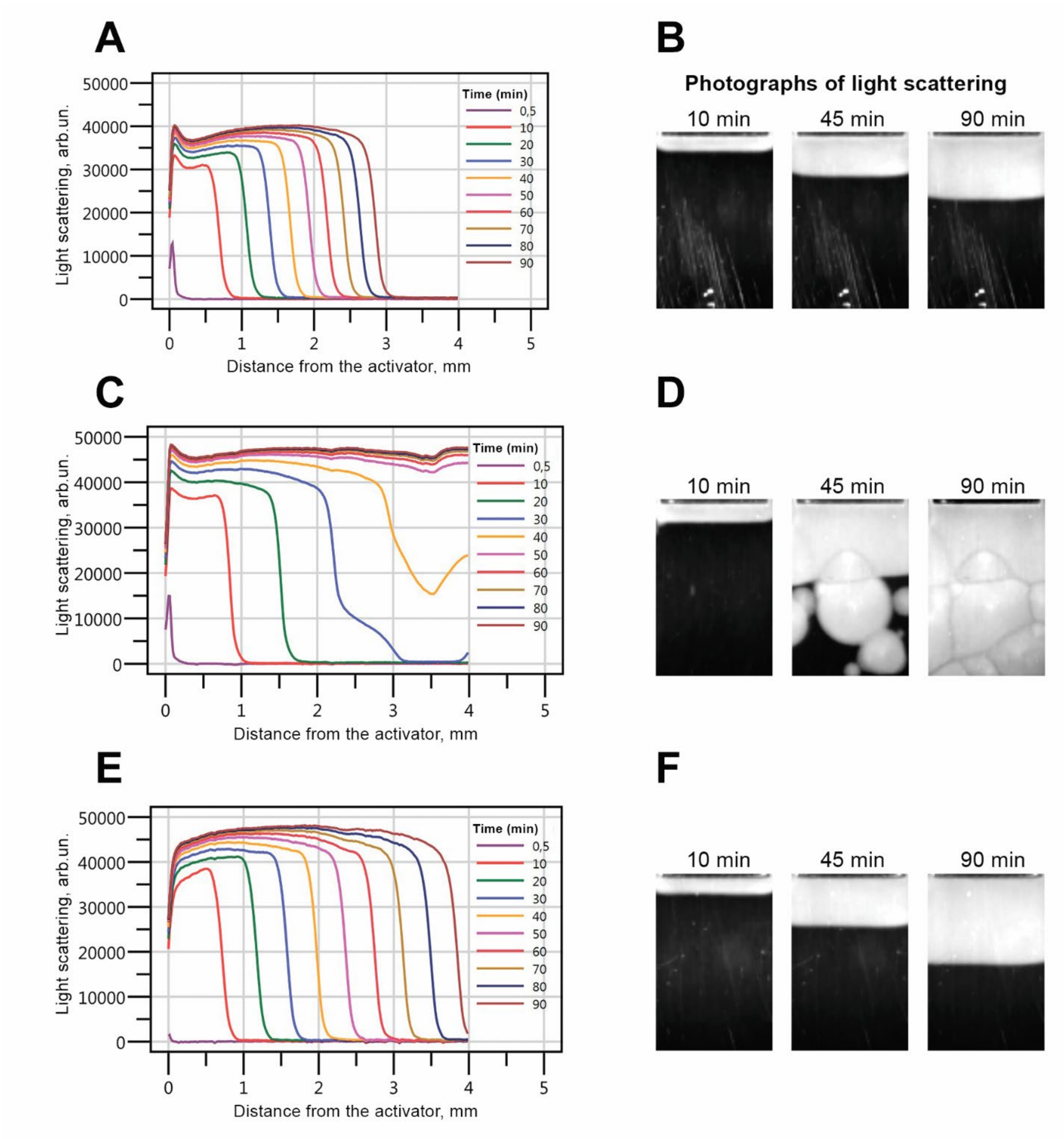

Figure S7. A typical example of coagulation dynamics in a patient receiving rivaroxaban (10 mg/day): one hour before surgery (panels **A**, **B**), after surgery but before taking the anticoagulant (panels **C** and **D**), and 3 hours after taking the anticoagulant (panels **E** and **F**). Panels **A**, **C** and **E** show the light scattering profiles obtained for these samples, and panels B, D and F show photographs of the corresponding clots 10, 45 and 90 min after the start of the experiment. Spontaneous clots are visible in the plasma sample after surgery (at 45 and 90 min) (panel **D**).

References

1 Eriksson BI, Dahl OE, Huo MH, et al.. Oral dabigatran versus enoxaparin for thromboprophylaxis after primary total hip arthroplasty (RE-NOVATE II*). A randomised, double-blind, non-inferiority trial. Thromb Haemost 2011; 105: 721-729

2 Eriksson BI, Borris LC, Friedman RJ, et al. Rivaroxaban versus enoxaparin for thromboprophylaxis after hip arthroplasty. N Engl J Med 2008; 358: 2765-2775

3 Rostin M, Montastruc JL, Houin G, D'Azemar P, Bayrou B, Boneu B. Pharmacodynamics of CY 216 in healthy volunteers: inter-individual variations. Fundam Clin Pharmacol 1990; 4: 17-23

4 Jiang S, Du L, Ni C. Comparing the efficacy, safety and cost of the anticoagulants: rivaroxaban and nadroparin in hip replacement surgery. Int J Pharmacol.2018; 14: 1-8

5 Stangier J. Clinical pharmacokinetics and pharmacodynamics of the oral direct thrombin inhibitor dabigatran etexilate. Clin Pharmacokinet 2008; 47: 285–295

6 Kubitza D, Becka M, Voith B, Zuehlsdorf M, Wensing G. Safety, pharmacodynamics, and pharmacokinetics of single doses of BAY 59-7939, an oral, direct factor Xa inhibitor. Clin Pharmacol Ther 2005; 78: 412-421

7 Mueck W, Stampfuss J, Kubitza D, Becka M. Clinical pharmacokinetic and pharmacodynamic profile of rivaroxaban. Clin Pharmacokinet 2014; 53: 1–16

8 Davis R, Faulds D. Nadroparin calcium. A review of its pharmacology and clinical use in the prevention and treatment of thromboembolic disorders. Drugs Aging 1997; 10: 299-322

9 Trocóniz IF, Tillmann C, Liesenfeld K.-H, Schäfer H-G, Stangier J. Population pharmacokinetic analysis of the new oral thrombin inhibitor dabigatran etexilate (BIBR 1048) in patients undergoing primary elective total hip replacement surgery. J Clin Pharmacol 2007; 47: 371–382

10 Hirsh J, Anand SS, Halperin JL, Fuster V, American Heart Association. Guide to anticoagulant therapy: Heparin. A statement for healthcare professionals from the American Heart Association. Circulation 2001; 103: 2994–3018

11 Lipets E, Vlasova O, Urnova E, et al. Circulating contact-pathway-activating microparticles together with factors IXa and XIa induce spontaneous clotting in plasma of hematology and cardiologic patients. PLoS One 2014; 9: e87692. Available at: https://journals.plos.org/plosone/article/file?id=10.1371/journal.pone.0087692&type=printable. Accessed November 2, 2024

12 Gribkova I, Lipets E, Rekhtina I, et al. The modification of the thrombin generation test for the clinical assessment of dabigatran etexilate efficiency. Sci Reports 2016; 6: 29242; Available at: https://pmc.ncbi.nlm.nih.gov/articles/PMC4932519/pdf/srep29242.pdf. Accessed November 2, 2024

13 Sinauridze EI, Vuimo TA, Tarandovskiy ID, et al. Thrombodynamics, a new global coagulation test: measurement of heparin efficiency. Talanta 2018; 180: 282-291

14 Koltsova EM. Kuprash AD, Dashkevich NM, et al. Determination of fibrin clot growth and spatial thrombin propagation in the presence of different types of phospholipid surfaces. Platelets 2021; 32: 1031-1037

15 Dashkevich NM, Ovanesov MV, Balandina AN, et al. Thrombin activity propagates in space during blood coagulation as an excitation wave. Biophys J 2012, 103: 2233–2240.
